# Translational readthrough of nonsense mutant TP53 by mRNA incorporation of 5-Fluorouridine

**DOI:** 10.1038/s41419-022-05431-2

**Published:** 2022-11-25

**Authors:** Mireia Palomar-Siles, Angelos Heldin, Meiqiongzi Zhang, Charlotte Strandgren, Viktor Yurevych, Jip T. van Dinter, Sem A. G. Engels, Damon A. Hofman, Susanne Öhlin, Birthe Meineke, Vladimir J. N. Bykov, Sebastiaan van Heesch, Klas G. Wiman

**Affiliations:** 1 Department of Oncology-Pathology, Karolinska Institutet, Stockholm, Sweden; 2 Princess Máxima Center for Pediatric Oncology, Utrecht, The Netherlands; 3 Department of Medical Biochemistry and Biophysics, Karolinska Institutet, Stockholm, Sweden

**Keywords:** Cancer therapy, Tumour-suppressor proteins, Cancer therapy, Tumour-suppressor proteins

## Abstract

*TP53* nonsense mutations in cancer produce truncated inactive p53 protein. We show that 5-FU metabolite 5-Fluorouridine (FUr) induces full-length p53 in human tumor cells carrying R213X nonsense mutant *TP53*. Ribosome profiling visualized translational readthrough at the R213X premature stop codon and demonstrated that FUr-induced readthrough is less permissive for canonical stop codon readthrough compared to aminoglycoside G418. FUr is incorporated into mRNA and can potentially base-pair with guanine, allowing insertion of Arg tRNA at the *TP53* R213X UGA premature stop codon and translation of full-length wild-type p53. We confirmed that full-length p53 rescued by FUr triggers tumor cell death by apoptosis. FUr also restored full-length p53 in *TP53* R213X mutant human tumor xenografts in vivo. Thus, we demonstrate a novel strategy for therapeutic rescue of nonsense mutant *TP53* and suggest that FUr should be explored for treatment of patients with *TP53* nonsense mutant tumors.

## Introduction

A significant fraction of cancer-associated loss of function mutations in tumor suppressor genes are nonsense mutations [1] that give rise to premature termination codons (PTCs) and truncated inactive proteins [2]. The tumor suppressor p53, encoded by the *TP53* gene, is a DNA-binding transcription factor that responds to cellular stress and regulates processes such as cell cycle arrest, apoptosis, senescence, and metabolism [3, 4]. *TP53* is the most frequently mutated gene in human tumors [5]. While the majority of *TP53* mutations in cancer are missense mutations that result in substitution of a single amino acid residue in the p53 protein, 10% of *TP53* mutations are nonsense mutations [6, 7] that result in truncated and functionally inactive p53. R213X is the most common *TP53* nonsense mutation in human tumors, and is one of the 10 most common somatic *TP53* mutations overall [6–8].

Aminoglycoside antibiotics such as G418 have been shown to induce translational readthrough of nonsense mutations in various genes including *TP53* [9]. However, their clinical use as readthrough-inducing agents is limited by nephrotoxicity [10] and ototoxicity [11]. We previously found that combination of aminoglycosides with Mdm2 inhibitors or proteasome inhibitors can further enhance full-length p53 levels in cells carrying R213X nonsense mutant *TP53* (ref. [12]), suggesting combination treatment strategies with lower doses of aminoglycosides. Previous studies have also identified novel readthrough-inducing compounds, including ELX-02 (NB124) [13, 14], Ataluren (PTC124/Translarna) [15], RTC13 and RTC14 (ref. [16]), clitocine [17], minor gentamicin complex component X2 (ref. [18]) 2,6-Diaminopurine [19] and SRI-41315 (ref. [20]). Ataluren has obtained conditional approval for treatment of Duchenne muscular dystrophy and ELX-02 (NB124) is being tested in phase 2 clinical trials in cystic fibrosis patients with nonsense mutations in the cystic fibrosis transmembrane conductance regulator (CFTR) gene (https://www.eloxxpharma.com/clinical-trials/).

Aminoglycosides have been suggested to induce readthrough by binding to the decoding center of the ribosome [21–23]. Various mechanisms have been proposed for other readthrough-inducing compounds, including mRNA incorporation [17], tRNA methylation inhibition [19] and reduction of eukaryotic release factor 1 (eRF1) abundance [20]. Importantly, PTCs appear more amenable for translational readthrough than normal termination codons at the 3’ end of coding sequences [24, 25], arguing that pharmacological induction of translational readthrough of disease-associated nonsense mutations should not necessarily affect normal termination of translation to any major extent.

Here we analyzed data from the NCI-60 database (https://dtp.cancer.gov/discovery_development/nci-60/) to identify compounds that preferentially target human tumor cells carrying nonsense mutant *TP53*. We identified 5-Fluorouracil (5-FU) as a potential readthrough-inducing agent. 5-FU is a well-known anticancer drug widely used for treatment of e.g. colorectal and breast cancer [26–28]. Cytotoxicity of 5-FU is due to its metabolites 5-Fluoro-2’-deoxyuridine (FdUr), which is incorporated into DNA and inhibits thymidylate synthase, and 5-Fluorouridine (FUr), which is incorporated into RNA [29], affecting pathways related to RNA modification and processing [28, 30]. We show that the readthrough effect of 5-FU is exerted by FUr, presumably via incorporation into mRNA. Ribo-seq analysis indicated that FUr induced translational readthrough of R213X PTC in *TP53* mRNA to a similar level as the aminoglycoside G418. FUr also increased full-length p53 levels in *TP53* R213X mutant human cancer cells in vivo in a mouse xenograft model. Finally, FUr-induced full-length p53 was transcriptionally active and triggered p53-dependent cell death in human cancer cells carrying R213X nonsense mutant *TP53*. These results raise the possibility that tumors carrying nonsense mutant *TP53* may be more sensitive to treatment with 5-FU and suggest that FUr itself should be explored as a therapeutic agent for such tumors.

## Results

### Identification of 5-FU as a potential nonsense mutant TP53 readthrough-inducing agent

**Fig. 1:**
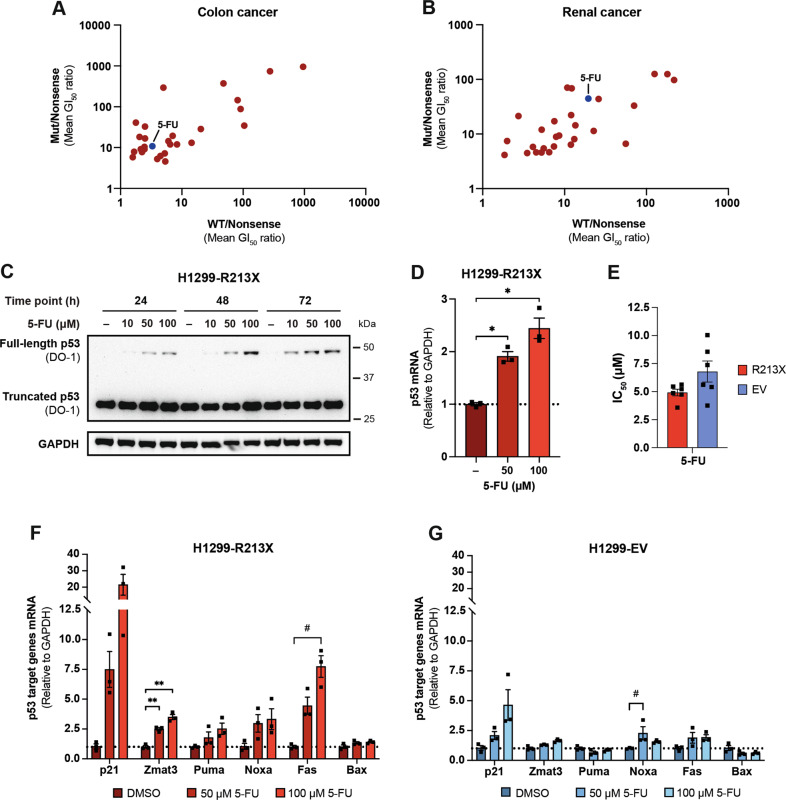
5-FU induces full-length and transcriptionally active p53. Ratios of mean 50% growth-inhibitory concentrations (GI_50_) for 28 top hit compounds between cancer cell lines carrying other *TP53* mutations and cancer cell lines carrying nonsense *TP53* mutations shown on the y-axis (Mut/Nonsense), and between cancer cell lines carrying WT *TP53* and cancer cell lines carrying nonsense *TP53* mutations shown on the x-axis (WT/Nonsense) in colon cancer cell lines **(A)** and renal cancer cell lines **(B)**. Each dot represents one compound. **C**, Full-length p53 induction by 5-FU in H1299-R213X cells is dose-dependent after 24, 48 or 72 h treatment, according to Western blotting with p53 antibody DO-1. GAPDH was used as loading control and DMSO (-) was used as negative control. Full membrane was blotted with DO-1 antibody, washed and blotted with GAPDH antibody. **D**, qRT-PCR analysis showing induction of p53 mRNA levels after 50 or 100 µM 5-FU treatment for 72 h in H1299-R213X cells, *N* = 3. DMSO (-) was used as negative control. **E**, IC_50_ (µM) values of 5-FU treatment for 72 h in H1299-R213X and H1299-EV cells, *N* = 6. **F**, mRNA levels of p53 target genes p21, Zmat3, Puma, Noxa, Fas and Bax after 50 or 100 µM 5-FU treatment for 72 h measured by qRT-PCR in H1299-R213X cells and **G**, H1299-EV cells, *N* = 3. In **D**, **F** and **G**, expression of some genes was examined simultaneously in each cell line with a single GAPDH control; thus, same GAPDH value was used as control for several genes. Gene expression values were normalized to GAPDH expression and to the DMSO-treated sample as negative control. Statistical analyses for each gene were performed comparing each treatment to DMSO control treatment using repeated measures one-way ANOVA followed by Dunnett’s multiple comparisons test (*p ≤ 0.05, **p ≤ 0.01) or Friedman test followed by Dunn’s multiple comparisons test (^#^*p* ≤ 0.05) in genes with data not fitting normal distribution. In **D**, **E**, **F** and **G**, data are represented as mean ± SEM. Each dot represents an independent experiment.

We performed data mining of the NCI-60 Human Tumor Cell Lines Screen (Developmental Therapeutics Program) in order to find compounds that preferentially suppress growth of *TP53* nonsense mutant tumor cells as compared to tumor cells carrying other *TP53* mutations or wild-type (WT) *TP53*. We extracted mean 50% growth-inhibitory concentrations (GI_50_) for 47 000 compounds in colon and renal cancer cell lines (Fig. S1A). Colon and renal cancer cell lines were chosen since the NCI-60 panel included lines with either WT, missense or nonsense mutant *TP53* for these tumor types. According to the COSMIC database, nonsense mutations account for 8.99% and 10.55% of all somatic *TP53* mutations in large intestine and renal cancer, respectively [8]. Based on the GI_50_ value for each compound and a simple algorithm (Fig. S1B), we identified 28 compounds with preferential effect on nonsense mutant *TP53* tumor cells (Fig. 1A-B). Among these, the clinically used anticancer drug 5-FU was selected for further study as a potential readthrough-inducing compound.

We first tested the ability of 5-FU to induce expression of full-length p53 protein in H1299 cells stably transfected with an R213X nonsense mutant *TP53* construct (H1299-R213X). 5-FU induced full-length p53 in a dose- and time-dependent manner according to Western blotting (Fig. 1C). In addition, qRT-PCR revealed a significant increase in *TP53* mRNA levels in H1299-R213X cells (Fig. 1D).

### Full-length p53 induced by 5-FU is transcriptionally active

In order to examine if full-length p53 induced by 5-FU retains specific DNA binding activity and capacity for transcriptional transactivation of target genes, we treated H1299-R213X cells and their empty vector equivalent (H1299-EV) with 5-FU and assessed p53 target gene levels by qRT-PCR. The half-maximal inhibitory concentration (IC_50_) for 5-FU was lower for H1299-R213X cells than H1299-EV cells (*p* = 0.087) (Fig. 1E). 5-FU at 100 µM resulted in an upregulation of both p21 (*p* = 0.1196) and Fas (*p* = 0.0286) mRNA in H1299-R213X cells (Fig. 1F). Zmat3 (Wig-1) mRNA was also significantly induced. Noxa and Puma were upregulated as well but this effect was not statistically significant. p21 and Noxa were also induced by 5-FU in H1299-EV cells, but to a lesser extent than in H1299-R213X cells (Fig. 1G). These results strongly suggest that the full-length p53 protein induced by 5-FU is at least partially functional with respect to DNA binding and transactivation of p53 target genes.

### 5-FU metabolite FUr is a potent inducer of full-length p53

**Fig. 2:**
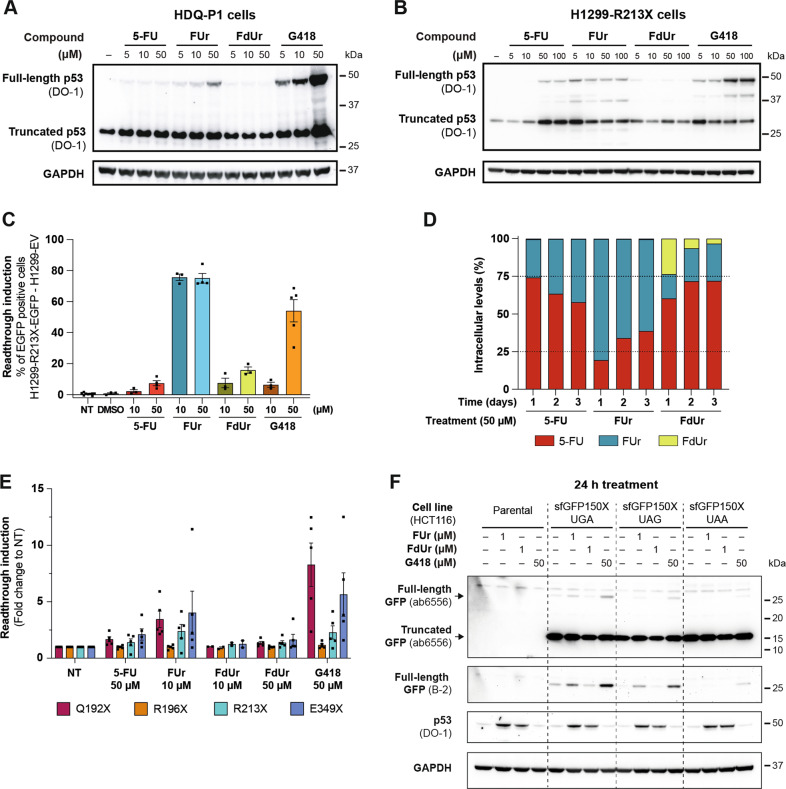
5-FU metabolite FUr rescues nonsense mutant TP53 and sfGFP150X. **A**, Western blot analysis of HDQ-P1 cells treated with 5, 10 or 50 µM 5-FU, FUr, FdUr or G418 for 72 h. Full-length p53 is induced in a dose-dependent manner mainly by FUr and G418. **B**, Western blot analysis of H1299-R213X cells treated with 5, 10, 50 or 100 µM 5-FU, FUr, FdUr or G418 for 72 h. **C**, *TP53* R213X readthrough induction measured by percentage of EGFP positive H1299-R213X-EGFP cells (the percentage of EGFP positive cells in H1299-EV control cells was subtracted). EGFP signal was detected by flow cytometry. Cells were treated with 10 or 50 µM 5-FU, FUr, FdUr or G418 for 72 h, *N* = 3-5. *N* = 3 for DMSO-treated control and *N* = 9 for non-treated control (NT). **D**, Intracellular levels of 5-FU, FUr or FdUr measured by LC-MS after treatment with 50 µM of each compound for 1, 2 or 3 days. Data are expressed as percentage of total levels of the three compounds per day. **E**, ELISA analysis of readthrough induction in H1299-R213X-ΔC-FLAG cells carrying *TP53* nonsense mutations Q192X, R196X, R213X or E349X after 5-FU, FUr, FdUr or G418 treatment for 72 h. ELISA plates were coated with anti-FLAG antibody to capture full-length p53 and amount of p53 was quantified with DO-1 antibody. Fold change of each sample to non-treated (-) control was calculated for each independent experiment, *N* = 2-5. **F**, Western blot analysis of WT *TP53* sfGFP150X HCT116 cells carrying a UGA, UAG or UAA PTC at sfGFP codon 150 after 24 h treatment with FUr, FdUr or G418. Full-length GFP was detected with GFP antibody B-2 and truncated GFP with GFP antibody ab6556, WT p53 stabilization was assessed with p53 antibody DO-1. GAPDH was used as loading control and untreated cells (-) as negative control. Membrane was blotted with GFP antibody ab6556, washed and blotted with GFP antibody B-2 to improve detection of the full-length GFP. Membrane was washed and blotted with DO-1 antibody, washed again and blotted with GAPDH antibody. In **A** and **B**, full-length and truncated p53 were detected with p53 antibody DO-1. GAPDH was used as loading control and DMSO (-) was used as negative control. Full membrane was blotted with DO-1 antibody, washed and blotted with GAPDH antibody. In **C** and **E**, each dot represents an independent experiment. Data are represented as mean ± SEM.

To study the mechanism by which 5-FU induces full-length p53 in R213X mutant *TP53* cells, we assessed readthrough induction by the two main 5-FU metabolites 5-Fluorouridine (FUr) and 5-Fluoro-2’deoxyuridine (FdUr). We first used HDQ-P1, a human breast carcinoma cell line that carries endogenous R213X nonsense mutant *TP53* (ref. [31]). FUr induced full-length p53 in these cells while FdUr had little or no effect (Fig. 2A). These results were confirmed in H1299-R213X cells in which FUr again was more potent than 5-FU (Fig. 2B). The strongest induction of full-length p53 was obtained with FUr at 5 µM whereas higher concentrations were less efficient, presumably due to substantial cell death. FdUr had little or no detectable effect on full-length p53 levels. G418, used as a positive control, induced full-length p53 with higher potency than FUr.

We also assessed *TP53* mRNA levels in H1299-R213X and HDQ-P1 cells upon treatment with FUr, FdUr or G418 (Fig. S2A-S2B). In H1299-R213X cells, we observed the strongest upregulation of *TP53* mRNA levels following treatment with 5 µM FUr, and modest upregulation also with FdUr and G418 (Fig. S2A). In HDQ-P1 cells, FUr did not induce *TP53* mRNA levels, while increased levels were observed after treatment with FdUr and G418. The increase was statistically significant only for G418 (Fig. S2B).

To further study induction of readthrough by 5-FU and its metabolites, we assessed their effects in H1299 cells stably transfected with *TP53* R213X cDNA fused in frame with EGFP (H1299-R213X-EGFP) by flow cytometry. Consistent with our previous results, FUr was the most potent *TP53* R213X readthrough inducer among 5-FU, FUr and FdUr (Fig. 2C). Already at 10 µM FUr treatment, more than 75% of cells were EGFP-positive while 5-FU and FdUr induced less than 20% EGFP-positive cells. G418 caused a marked increase in EGFP-positive cells at 50 µM but was still less potent than FUr (Fig. 2C). We validated our flow cytometry results by Western blotting (Fig. S2C). Thus, the readthrough effect observed with 5-FU is most likely due to conversion to FUr. Indeed, we detected intracellular FUr already one day after 5-FU treatment and an even higher fraction after two and three days (Fig. 2D). FUr entered the cells more efficiently than 5-FU and FdUr (Table S1).

We then tested the ability of 5-FU, FUr and FdUr to stimulate readthrough of different common *TP53* nonsense mutants using ELISA and H1299 cells stably transfected with constructs carrying the *TP53* coding sequence up until the PTC to be tested, followed by a FLAG tag (Fig. 2E). FUr was most potent for E349X, followed by Q192X, R213X and R196X. The same order of readthrough efficiency was observed for 5-FU, although 5-FU was less potent. FdUr did not have any major readthrough effect. G418 induced the highest readthrough of Q192X, followed by E349X, R213X and R196X.

### FUr rescues nonsense mutations in other genes than TP53

To test whether FUr can induce translational readthrough in a non-*TP53* context, we used *TP53* WT HCT116 colon carcinoma cells stably transfected with superfolder GFP with a UGA, UAG or UAA PTC at position 150 (HCT116 sfGFP150X). FUr treatment resulted in expression of full-length GFP in cells carrying sfGFP150X with a UGA or UAG but not UAA (Fig. 2F). G418 induced full-length sfGFP levels most efficiently in cells carrying sfGFP with UGA, less potently in cells with UAG, and only to a very minor extent in cells with UAA. FdUr had no detectable effect. We also treated three different HCT116 sublines stably transfected with WT sfGFP. Western blotting showed that FUr did not stabilize WT sfGFP in any of the sublines (Fig. S2D). In addition, we assessed readthrough induction by FUr in H1299 cells stably transfected with an EGFP reporter construct in which the coding sequence of EGFP is followed by a UGA stop codon and a C-terminal FLAG tag (H1299-EGFP-X-FLAG). The nine nucleotides upstream and downstream of the stop codon are identical to those surrounding the PTC in the *TP53* R213X mutant to mimic the sequence context in *TP53*. Both FUr and G418 induced readthrough as detected by anti-FLAG antibody (Fig. S2E). Low levels of full-length protein were also detected in untreated cells, presumably due to spontaneous basal readthrough.

These results show that FUr can induce stop-codon readthrough in nonsense mutant genes other than *TP53*, supporting our hypothesis that it has genuine translational readthrough activity as opposed to mere stabilization of full-length protein produced by basal readthrough. Nonetheless, FUr and FdUr induced WT p53 in all HCT116 sublines (Fig. 2F; Fig. S2D), in agreement with previous studies showing that 5-FU can stabilize WT p53 [32–34]. Thus, FUr may increase full-length p53 protein levels in R213X mutant *TP53* cells through a dual mechanism that involves both induction of translational readthrough and p53 protein stabilization (see further below).

### Ribosome profiling supports induction of translational readthrough by FUr

**Fig. 3:**
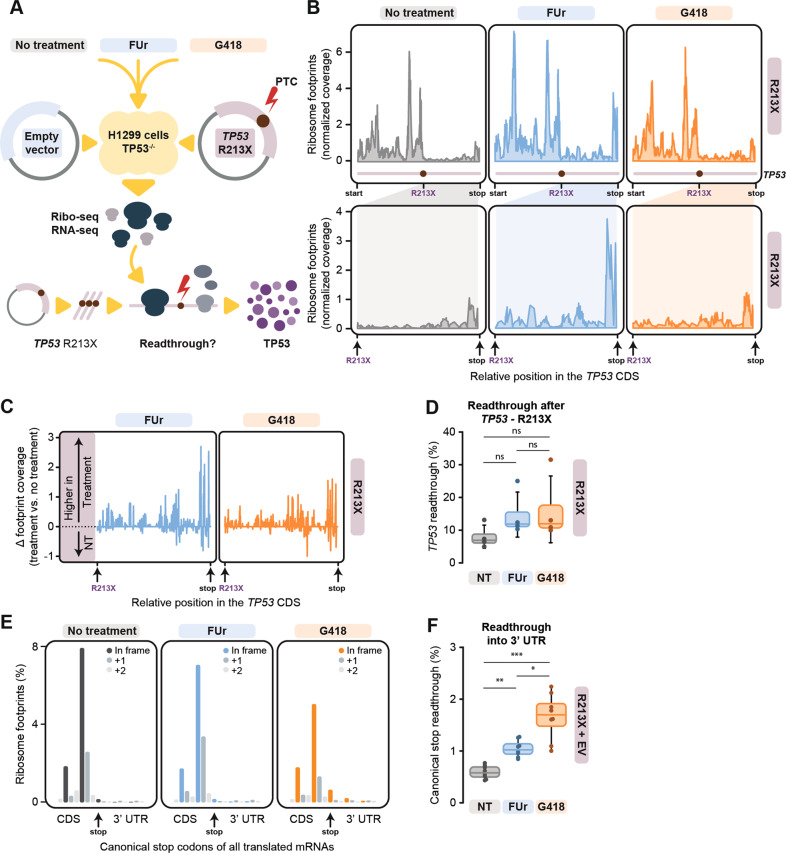
Ribosome profiling supports induction of translational readthrough by FUr. **A**, Schematic overview of the Ribo-seq experiments. **B**, Line plots of normalised Ribo-seq read coverage across the entire *TP53* CDS (top) and a zoomed view of the region between R213X and the canonical *TP53* stop codon (bottom). Locations of start and stop positions and premature termination codon R213X are annotated. **C**, Segment plots of relative Ribo-seq read coverage of *TP53* between the premature termination codon R213X and the canonical stop codon. Positive and negative values indicate coverage changes in H1299-R213X cells upon treatment with either FUr or G418 in comparison to untreated cells. **D**, Box plot of premature R213X stop codon readthrough ratios observed by Ribo-seq. Ratios were calculated by taking the sum of all in-frame reads after the R213X premature stop until the canonical stop codon and dividing it with the sum of all in-frame reads from the canonical start position until the premature stop codon, normalized for CDS codon length (*see Methods*), *N* = 4. Statistical analyses were performed comparing all conditions to each other using Friedman test followed by Dunn’s multiple comparisons test as data did not fit normal distribution (ns = not significant). **E**, Metagene bar plot showing footprint coverage around the stop codons of annotated reading frames of all translated protein-coding genes. **F**, Box plot of stop codon readthrough ratios on a metagene level, as determined by Ribo-seq per treatment condition. Ratios were calculated by taking the sums of in-frame reads after the stop codon and dividing these with the sums of in-frame reads before the stop codon, both normalised for feature length. Replicate experiments in H1299-EV and H1299-R213X cells were merged per treatment type for this visualization, *N* = 8. Statistical analyses were performed comparing all conditions to each other using repeated measures one-way ANOVA followed by Tukey’s multiple comparisons test (*p ≤ 0.05, **p ≤ 0.01, ***p ≤ 0.001). In **D** and **F**, each dot represents a replicate experiment. Data are represented as median ± 95% confidence interval, box limits represent the upper and lower quartiles.

To investigate FUr-induced translational readthrough of R213X mutant *TP53* at nucleotide resolution, we performed Ribo-seq analysis (Fig. 3A and Fig. S3A–C). G418, previously shown to induce translational readthrough by Ribo-seq [25], was used as positive control. Sequenced Ribo-seq reads displayed expected size distributions around 28-29 nucleotides (nt) (Fig. S3D) and high 3-nt codon periodicity (Fig. S3E-F), resulting in ribosome footprints primarily aligning to annotated coding sequences (CDSs; 91%; min 86% - max 95%).

We could visualize translation across the entire coding sequence of *TP53*, with translational readthrough at R213X visibly enhanced by both FUr and G418 (Fig. 3B). This effect became more apparent when normalized for ribosome occupancy post-R213X in untreated cells (Fig. 3C), with an increased median readthrough ratio of 11.86% and 11.94% for FUr and G418, respectively, as compared to 6.9% for untreated cells (Fig. 3D), and as defined by the ratio of the normalized occupancy of Ribo-seq reads after versus before the R213X mutation (*see Methods*). Although statistical significance was not reached, the results indicated an increased readthrough after both FUr and G418 treatments (Fig. 3D). We next investigated whether FUr would induce readthrough at canonical translation termination codons at the ends of annotated CDSs, as previously shown for G418 (ref. [25]). For both compounds, readthrough at canonical stop codons was significantly enhanced over background (i.e., no treatment) as judged by the percentage of in-frame reads after the annotated stop codon (medians of 0.58% for no treatment; 1.04% for FUr; 1.7% for G418), although levels were much lower than those at PTCs (Fig. 3E-F). This statistically validated the status of FUr as a readthrough agent and shows that FUr-induced readthrough was more restricted to PTCs and less pronounced at canonical stops than canonical readthrough induced by G418 (*p* = 0.0222; Fig. 3F), possibly indicative of lower rates of pan-translatome perturbations.

### FUr induces R213X p53-dependent cell death

**Fig. 4:**
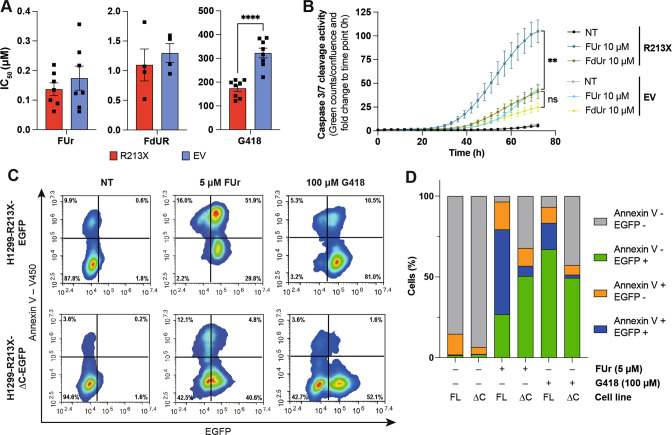
FUr induces R213X p53-dependent cell death. **A**, IC_50_ (µM) values of FUr, FdUr or G418 treatment for 72 h in H1299-R213X and H1299-EV cells. Each dot represents an independent experiment, *N* = 4-8. Differences between the two cell lines within each treatment group were compared using independent *t*-test, ****p ≤ 0.0001. **B**, Caspase 3/7 cleavage activity in H1299-R213X and H1299-EV cells after treatment with 10 µM FUr or FdUr up to 72 h, *N* = 3-4. Differences between FUr-treated H1299-R213X vs. H1299-EV cells (*p* = 0.0029) or between FdUr-treated H1299-R213X vs. H1299-EV cells at 72 h treatment time point were analyzed using independent *t*-test. **C**, Plots of a representative experiment of Annexin V staining and EGFP expression as assessed by flow cytometry in H1299-R213X-EGFP and H1299-R213X-ΔC-EGFP cells after 5 µM FUr or 100 µM G418 treatment for 72 h. See also Fig. S4D for the other two experiment replicates. **D**, Quantification of 3 independent experiments represented in Fig. 4C and Fig. S4D. Mean percentage of Annexin V negative and EGFP negative (grey bars), Annexin V negative and EGFP positive (green bars), Annexin V positive and EGFP negative (orange bars) and Annexin V positive and EGFP positive (blue bars) H1299-R213X-EGFP (FL) or H1299-R213X-ΔC-EGFP (ΔC) cells after treatment with 5 µM FUr or 100 µM G418 for 72 h. *N* = 3. In **A** and **B**, data are represented as mean ± SEM.

Next, we asked whether induction of full-length p53 by FUr would trigger a biological response. H1299-R213X cells are more sensitive to FUr and FdUr than H1299-EV cells as assessed by WST-1 (Fig. 4A), but these differences are not statistically significant. We first assessed p53-induced apoptosis by monitoring caspase-3/7 activation over time using Incucyte®. Treatment with FUr triggered a significantly higher caspase 3/7 activity in H1299-R213X cells compared to H1299-EV at 72 h, whereas FdUr treatment did not (Fig. 4B). Cell morphology and caspase 3/7 activity after FUr or FdUr treatment is shown in Fig. S4A. A statistically significant difference in confluence was observed between H1299-R213X and H1299-EV cells after FUr treatment at 72 h (*p* = 0.0182) (Fig. S4B).

Second, we assessed apoptosis by Annexin V staining after treatment with FUr in H1299 cells stably transfected with a construct containing the p53 coding sequence up to the R213X PTC fused in-frame with the coding sequence of EGFP (H1299-R213X-ΔC-EGFP), or a construct containing the full-length p53 coding sequence with R213X fused in frame with the coding sequence of EGFP (H1299-R213X-EGFP). These two cell lines allowed us to determine both readthrough induction by assessing EGFP expression and p53-dependent biological effects induced by full-length p53 protein following readthrough. FUr induced readthrough in both H1299-R213X-ΔC-EGFP and H1299-R213X-EGFP cells (Fig. 4C) which was validated by Western blotting (Fig. S4C). We observed a higher percentage of Annexin V-positive cells in the H1299-R213X-EGFP cells compared to the H1299-R213X-ΔC-EGFP control cells, indicating a p53-dependent apoptotic effect. G418 induced readthrough in both cell lines but was less efficient than FUr in inducing apoptosis according to Annexin V staining (Fig. 4D; Fig. S4D). A large fraction of H1299-R213X-EGFP cells underwent apoptosis (orange and blue bars) upon treatment with FUr. The predominant cell population (52.6%) was Annexin V+/ EGFP+ (blue bars). In contrast, only a minor fraction (6.2%) of the H1299-R213X-ΔC-EGFP cells were apoptotic and EGFP positive after FUr treatment. G418 potently induced EGFP expression (green and blue bars) in H1299-R213X-EGFP cells, but a more modest fraction of Annexin V+ cells (orange plus blue bars) and Annexin V+/EGFP+ cells (blue bars; 16.25%). We also assessed apoptosis in H1299-R213X cells and observed a higher fraction of Annexin V-positive cells compared to H1299-EV, parental H1299, and H1299-R213X-ΔC-FLAG cells after treatment with FUr (Fig. S4E). In HDQ-P1, FUr induced a robust and statistically significant increase in the fraction of Annexin V-positive cells whereas FdUr had a more modest effect (Fig. S4F).

Altogether, these results show that FUr-induced full-length p53 is capable of triggering cell death by apoptosis.

### FUr-induced full-length p53 is transcriptionally active

**Fig. 5:**
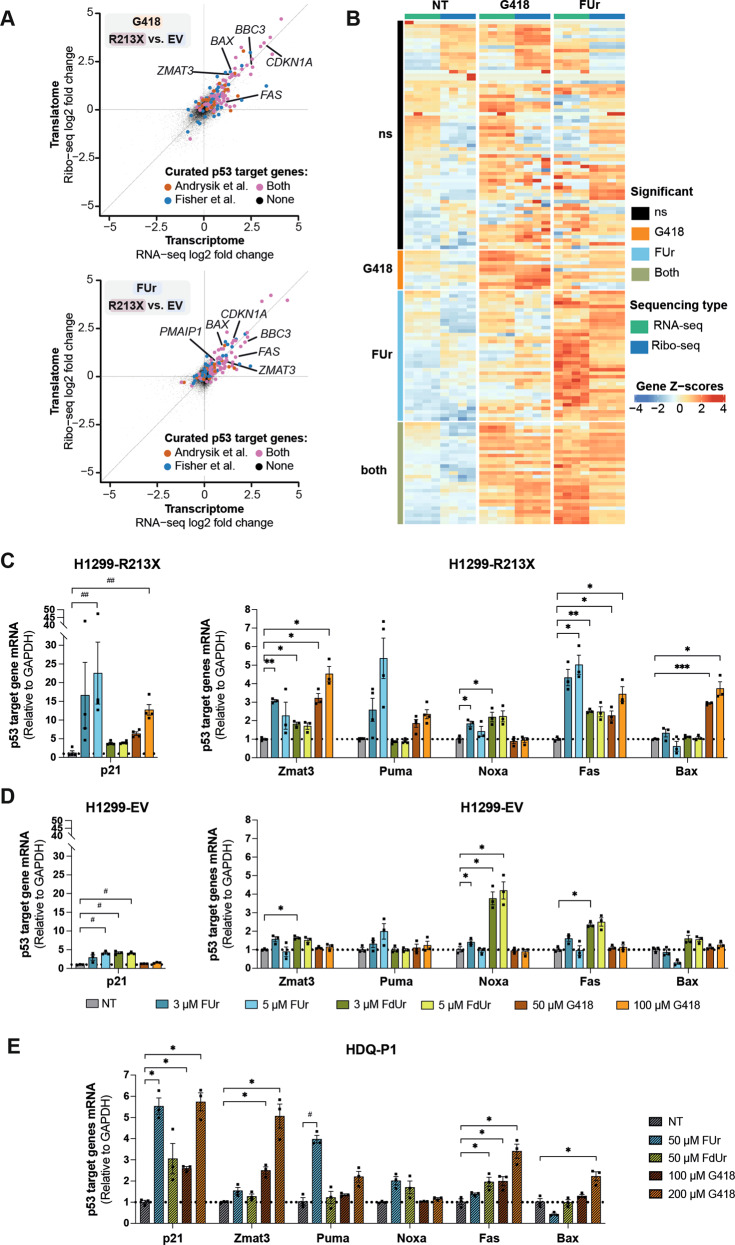
FUr-induced full-length p53 is transcriptionally active. **A**, RNA-seq to Ribo-seq log2 fold-change/fold-change (FC/FC) plots showing differentially transcribed (x axis) and translated (y axis) genes for G418 (top) and FUr (bottom) treatments. Each dot on each graph represents a protein-coding gene. Curated *TP53* target genes from Andrysik et al. [35] are coloured red and curated *TP53* target genes from Fischer [36] are in blue. Genes found in both lists are coloured purple. Selected *TP53* target genes validated by qRT-PCR in (**C** and **D**) are labelled by gene symbol, *CDKN1A* (p21), *ZMAT3* (Zmat3), *BBC3* (Puma), *PMAIP1* (Noxa), *FAS* (Fas) and *BAX* (Bax). **B**, Heatmap showing gene expression of *TP53* target gene lists as obtained from Andrysik et al. [35] and from Fischer [36], organized per treatment condition in H1299-R213X cells, as measured by RNA-seq (green, left) and ribo-seq (blue, right). Statistical analyses for each treatment were performed using DESeq2 (ref. [56]), i.e., a Wald test with Benjamin-Hochberg multiple test correction (adjusted *p*-value ≤ 0.05, log2 fold change ≥ 1) to test for significant genes in Ribo-seq data, colours indicate differentially expressed genes. Normalised counts were transformed to Z-scores for visualisation. **C**, mRNA levels of p53 target genes genes p21, Zmat3, Puma, Noxa, Fas and Bax after 3 or 5 µM FUr or FdUr, or 50 or 100 µM G418 treatment for 72 h measured by qRT-PCR in H1299-R213X cells and **D**, in H1299-EV cells. *N* = 3-4. **E**, mRNA levels of p53 target genes p21, Zmat3, Puma, Noxa, Fas and Bax after 50 µM FUr or FdUr, or 100 or 200 µM G418 treatment for 72 h measured by qRT-PCR in HDQ-P1 cells. *N* = 3. In **C**, **D** and **E**, data are represented as mean ± SEM. Values were normalized to GAPDH expression and to the non-treated sample (NT). Statistical analyses for each cell line and each gene were performed comparing each treatment to only each control non-treated (NT) sample using repeated measures one-way ANOVA followed by Dunnett’s multiple comparisons test (*p ≤ 0.05, **p ≤ 0.01, ***p ≤ 0.001) or Friedman test followed by Dunn’s multiple comparisons test (^#^*p* ≤ 0.05, ^##^*p* ≤ 0.01) in genes with data not fitting normal distribution. In **C**, **D, E**, Figure S2A and Figure S2B, expression of some genes was examined simultaneously in each cell line with a single GAPDH control; thus, same GAPDH value was used as control for several genes.

Ribo-seq and RNA-seq analysis allowed us to examine effects of FUr and G418 on the transcriptomes and translatomes of H1299-R213X cells. Both G418 and FUr induced p53-dependent transcription of p53 target genes (Fig. 5A). We observed pronounced effects on both the transcriptome and translatome after FUr treatment, and these effects were more drastic than those observed upon treatment with G418 (Fig. S5A). Gene expression changes induced by FUr and G418 were correlated to two manually curated p53 target gene lists published by Andrysik et al. [35] (Fig. S5B) and Fischer [36] (Fig. S5C). The two gene lists were merged in Fig. 5B. Several p53 target genes were significantly upregulated upon treatment with either G418 or FUr. However, the subset induced by FUr was larger than that induced by G418 (Fig. 5B).

To validate the RNA-seq results and further assess p53-dependent transcription, we examined mRNA levels of a panel of p53 target genes by qRT-PCR upon treatment with FUr, FdUr and G418. p21, Zmat3 and Fas mRNA were significantly upregulated by FUr in the H1299-R213X cells (Fig. 5C), with little or no increase in FUr-treated H1299-EV cells (Fig. 5D). Noxa showed a small but statistically significant upregulation by FUr in the H1299-R213X cells and minor increase in the H1299-EV cells. Bax was not upregulated by FUr in neither cell line. FdUr had relatively modest effects on mRNA levels for most of the p53 target genes and the effects were in general independent of p53. A robust induction of Noxa and Fas was observed in H1299-EV cells (Fig. 5D). These results are consistent with the fact that FUr but not FdUr induces significant levels of full-length p53 in the H1299-R213X cells (Fig. 2B). Thus, any effects of FdUr must be attributed to readthrough- and p53-independent mechanisms. G418 induced p21, Zmat3, Puma, Fas and Bax in H1299-R213X cells, which was statistically significant for all genes except Puma (Fig. 5C). FUr and G418 induced different patterns of p53 target gene expression in these cells. p21, Puma and Fas showed the strongest upregulation by FUr while Zmat3 and Bax were most potently induced by G418. p53 target gene expression was also examined in HDQ-P1 cells (Fig. 5E). We observed a statistically significant upregulation of p21 and Puma upon FUr treatment. This supports the conclusion that full-length p53 induced by FUr in these cells that carry endogenous R213X *TP53* has transcriptional transactivation activity.

In order to further validate the transcriptional activity of FUr-induced full-length p53, a construct with 13 p53 consensus DNA binding motifs upstream of EGFP was transfected into H1299-R213X cells prior to treatment with FUr, FdUr or G418. Full-length p53 induced by FUr or G418 caused a robust increase in expression of EGFP (Fig. S5D). In addition, the DNA-binding activity of full-length p53 was examined in the same cells by the TransAM p53 DNA binding assay. Both FUr and G418 induced full-length p53 that was capable of binding to a DNA oligo with a p53 consensus motif (Fig. S5E). A WT p53 DNA oligo but not a mutant oligo competed for FUr-induced p53 DNA binding, indicating that the DNA binding was specific.

### FUr is efficiently incorporated into mRNA

**Fig. 6:**
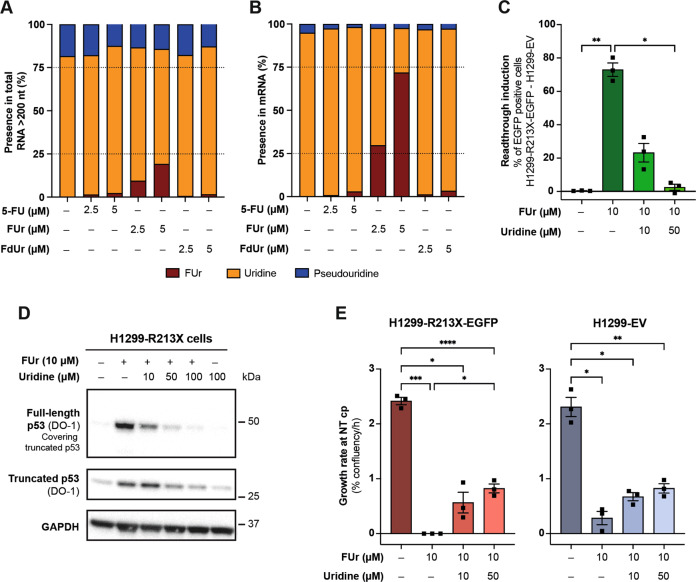
FUr is efficiently incorporated into mRNA. **A**, Content of FUr, uridine and pseudouridine as percentage of total content of FUr, uridine and pseudouridine after 5-FU, FUr or FdUr treatment for 3 days in total RNA species longer than 200 nucleotides (nt) and **B**, in mRNA. **C**, Flow cytometry analysis of readthrough induction after 10 µM FUr treatment alone or in combination with 10 or 50 µM uridine for 72 h. Readthrough induction was assessed by percentage of EGFP positive H1299-R213X-EGFP cells (EGFP positive cells in H1299-EV control cells were subtracted), *N* = 3. Note that results were obtained in two of the experiments presented in Fig. S6A, so values for two non-treated samples (NT) are the same in both figures. **D**, Western blot analysis of H1299-R213X cells treated with 10 µM FUr alone or in combination with 10, 50 or 100 µM uridine or 100 µM uridine alone for 72 h. Full membrane was incubated with DO-1 antibody to visualize truncated p53. Truncated p53 was then covered and the membrane was exposed again to show full-length p53 bands. Membrane was cut and blotted with GAPDH, which was used as loading control. **E**, Cell growth rate of H1299-R213X-EGFP or H1299-EV cells untreated or treated with 10 µM FUr alone or in combination with 10 or 50 µM uridine was monitored up to 72 h with Incucyte® S3 system. Data are presented as cell growth rate in percentage of confluency per hour when growth rate of the non-treated cell population (-) reaches its maximum or critical point (cp), *N* = 3. Note that results were obtained in two of the experiments presented in Fig. S6B, and so values for two non-treated samples (NT) are the same in both figures. In **C** and **E**, data are represented as mean ± SEM. Statistical analyses for each panel were performed comparing all conditions to each other using repeated measures one-way ANOVA followed by Tukey’s multiple comparisons test (*p ≤ 0.05, **p ≤ 0.01, ***p ≤ 0.001, ****p ≤ 0.0001).

It is conceivable that FUr-induced translational readthrough involves incorporation of FUr into RNA. To test this, we extracted total RNA longer than 200 bases, excluding 5.8 S rRNA, 5 S rRNA, tRNAs and small noncoding RNAs, from FUr-treated H1299-R213X cells, purified mRNA and analyzed both total RNA and mRNA for content of uridine, pseudouridine and FUr by LC-MS. The content of FUr reached 19.1% of the total content of FUr, uridine and pseudouridine in total RNA after treatment with 5 µM FUr (Fig. 6A) and 71.8% of the total FUr, uridine and pseudouridine content in mRNA (Fig. 6B).

Next, we performed competition experiments with uridine in H1299-R213X-EGFP cells. Flow cytometry demonstrated that combination of FUr with uridine at a 1:1 ratio led to a decrease in readthrough induction to about one third of the level observed with FUr alone, and at a ratio of 1:5, readthrough induction was almost completely blocked (Fig. 6C). In contrast, uridine only had a modest effect on readthrough induced by 10 µM of G418, and had no effect on the low level of readthrough induced by FdUr when combined at a 1:1 or 1:5 ratio (Fig. S6A). The decrease in readthrough induction at 50 µM G418 in combination with uridine at a 1:1 or 1:5 ratio was small and not statistically significant. Moreover, combination of FUr and uridine resulted in dose-dependent decrease in full-length p53 protein levels in H1299-R213X cells (Fig. 6D).

We then asked if combination treatment with uridine would affect FUr-induced inhibition of cell proliferation in H1299-R213X-EGFP and H1299-EV cells as assessed by Incucyte®. Treatment with FUr alone at 10 µM inhibited the growth of both cell lines (Fig. 6E). Combination of FUr with 10 or 50 µM uridine caused a partial rescue of cell proliferation in both cell lines. This effect was statistically significant at 10 µM FUr in combination with uridine at a 1:5 ratio in H1299-R213X-EGFP cells but not H1299-EV cells, suggesting that the rescue was related to decreased expression of full-length p53. Combination of FdUr with uridine at a ratio of 1:1 or 1:5 showed a slight rescue in cell proliferation in both H1299-R213X-EGFP and H1299-EV cells. Uridine alone did not have any impact on cell proliferation (Fig. S6B). These results indicate that FUr induces translational readthrough at least in part by incorporation into mRNA. Added uridine competes with FUr for incorporation into mRNA, which leads to decreased *TP53* R213X readthrough and alleviated FUr-induced inhibition of cell proliferation.

### 5-FU and FUr induce full-length p53 in vivo

**Fig. 7:**
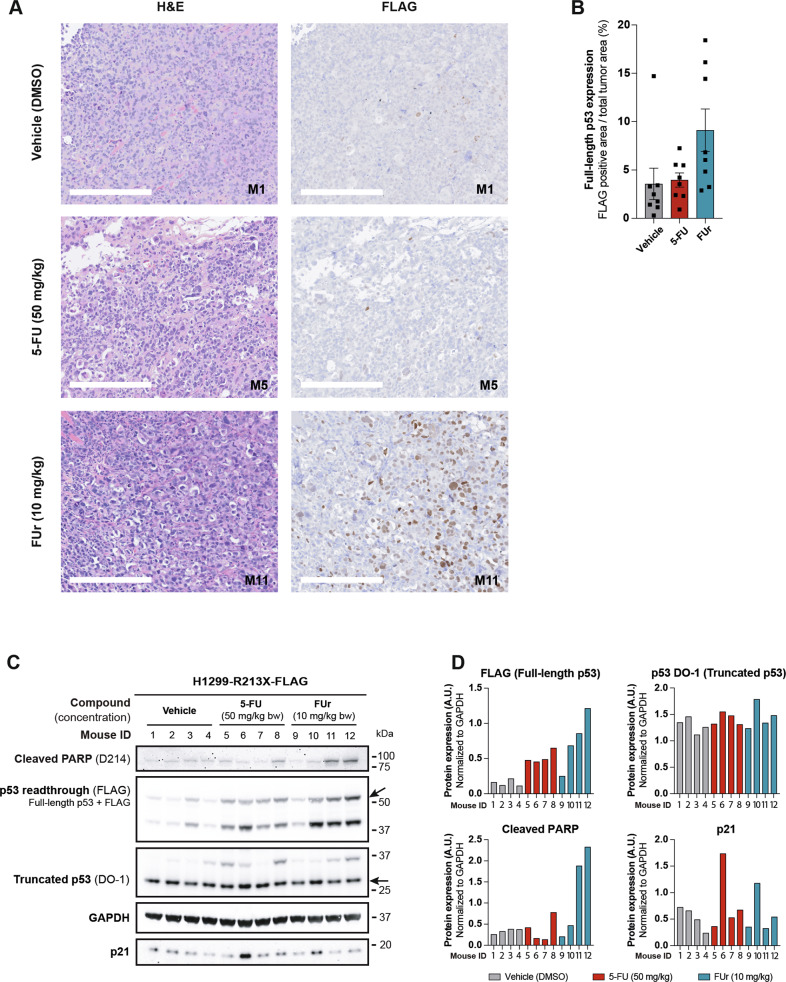
5-FU and FUr induce full-length p53 in vivo. **A**, Representative images of hematoxylin and eosin (H&E) staining and immunohistochemistry (IHC) of FLAG for full-length p53 detection in H1299-R213X-FLAG xenograft tumors from mice treated with 50 mg/kg of body weight (mg/kg bw) of 5-FU or 10 mg/kg bw of FUr for 5 days in one week. Scale bars = 250 µm. **B**, Quantification of FLAG staining in Fig. 7A. Data are presented as percentage of FLAG-positive area relative to all tumor area. *N* = 4 mice in each treatment group, and two sections from each mouse tumor were stained and quantified. Each dot corresponds to one section, data are represented as mean ± SEM. **C**, Western blot analysis of H1299-R213X-FLAG xenograft tumors from mice treated with 50 mg/kg bw of 5-FU or 10 mg/kg bw of FUr for 5 days in one week. Readthrough induction was detected with FLAG antibody and truncated p53 with p53 antibody DO-1. Cleaved PARP was included as an apoptotic marker and was detected with Cleaved PARP D214 antibody. The p53 target p21 was detected with p21 F-5 antibody. GAPDH was used as loading control. Membrane was cut at 75 and 25 kDa. Upper part was blotted with Cleaved PARP antibody. Middle part was blotted with FLAG antibody, then washed and blotted with DO-1 antibody, washed and blotted with GAPDH antibody. Bottom part was blotted with p21 antibody. Upper arrow indicates full-length p53 and lower arrow truncated p53. **D**, Western blot quantification of Fig. 7C. Data are presented as expression of each indicated protein relative to GAPDH expression. Each bar corresponds to one mouse.

To determine if 5-FU and FUr were capable of inducing full-length p53 in vivo, we inoculated H1299-R213X-FLAG and H1299-R213X-ΔC-FLAG cells in the right and left flank, respectively, of female athymic nude mice. The mice were treated i.p. with 5-FU or FUr once daily for 5 days. Xenograft tumors were analyzed by IHC and Western using FLAG antibody to detect the full-length p53-FLAG fusion protein. We observed elevated expression of p53-FLAG, especially in tumors from FUr-treated mice, according to IHC staining (Fig. 7A-B). Western blotting revealed that treatment with 5-FU or FUr for 5 days was sufficient to induce detectable levels of full-length p53 in vivo in H1299-R213X-FLAG xenografts (Fig. 7C-D). p53 expression, detected by FLAG antibody, was the highest in tumors from three of the FUr-treated mice (ID 10, 11 and 12), but was also elevated in tumors from 5-FU-treated mice. Detectable levels of full-length p53 in the vehicle-treated mice (ID 1, 2, 3 and 4) are presumably due to spontaneous readthrough. The second band above 37 kDa is most likely a degradation product of p53 (Fig. S7A).

We observed a detectable increase in *TP53* R213X readthrough also in H1299-R213X-ΔC-FLAG xenografts from two FUr-treated mice (ID 10 and 12) (Fig. S7B). Levels of truncated p53 were similar in all tumors and in both cell lines from both treated and vehicle-treated mice (Fig. 7C-D; Fig. S7B).

To assess activity of full-length p53 in the xenograft tumors, we examined protein levels of the apoptotic marker cleaved PARP and the p53 target gene p21 in the H1299-R213X-FLAG xenografts. Cleaved PARP was upregulated in the tumor from one mouse treated with 5-FU (mouse ID 8) and in tumors from two mice treated with FUr (mice ID 11 and 12). p21 showed increased expression in the tumor from one mouse (ID 6) treated with 5-FU and to a lesser extent in one mouse (ID 10) treated with FUr. Levels of cleaved PARP remained relatively similar in all H1299-R213X-ΔC-FLAG xenografts. However, p21 expression was slightly elevated in H1299-R213X-ΔC-FLAG xenografts in two mice treated with 5-FU and FUr (mice ID 8 and 10) (Fig. S7B).

All mice showed no or only minor weight loss during the 5 days treatment period (Fig. S7C). Average initial tumor volume of H1299-R213X-FLAG xenografts for the three groups were similar (Fig. S7D-E). Tumor volumes after the last treatment were on average larger in the vehicle-treated and 5-FU-treated mice compared to the FUr-treated mice for both types of xenografts (Fig. S7D-E).

## Discussion

Pharmacological reactivation of missense mutant p53 as a strategy for cancer therapy is already being tested in the clinic [37, 38]. In contrast, reactivation of *TP53* nonsense mutant tumors is still in its infancy as a therapeutic strategy. Aminoglycoside antibiotics have been shown to induce translational readthrough of nonsense mutant *TP53* and expression of full-length p53 that retains transcriptional transactivation activity [9, 12]. We analyzed data available at the NCI-60 database and identified the well-known anticancer drug 5-Fluorouracil (5-FU) as an agent with preferential toxicity in *TP53* nonsense mutant colon and renal cancer cells. We confirmed induction of full-length p53 protein by 5-FU in H1299 cells carrying exogenous R213X mutant *TP53* (H1299-R213X) and verified that the full-length protein was able to transactivate p53 target genes. Our further analysis of the two main 5-FU metabolites FdUr and FUr demonstrated that the induction of full-length p53 by 5-FU in R213X mutant *TP53* cells is largely mediated by FUr whereas FdUr has little activity. Moreover, FUr but not FdUr caused a strong induction of full-length GFP protein in HCT116 sfGFP150X cells carrying UGA or UAG PTC. Similarly, FUr induced readthrough in cells transfected with an EGFP reporter gene followed by a PTC and a FLAG tag. In vivo, we also observed a more potent induction of full-length p53 levels with FUr than with 5-FU in xenograft tumors with R213X *TP53* nonsense mutation. The lack of significant inhibition of H1299-R213X-FLAG xenograft tumor growth by FUr in this experiment could be due to the short duration of the treatment.

Our LC-MS data on cellular uptake are entirely consistent with FUr being the critical metabolite for induction of full-length p53. We found markedly higher intracellular concentrations of FUr as compared to FdUr after treatment with 5-FU (Table S1). However, conversion to FUr was not equimolar and the amount of FUr produced from 5-FU was well below FUr levels detected after treatment with FUr itself.

Analysis of Ribo-seq data demonstrated an increase in sequence reads 3’ of the R213X PTC in *TP53* mRNA upon treatment with FUr, providing further evidence for a translational readthrough effect. The efficiency of FUr-induced translational readthrough was similar to that of G418 at the concentrations tested, although G418 had a more prominent effect on translational readthrough at canonical termination codons than what we observed for FUr.

It is well known that 5-FU can stabilize WT p53 (refs. [32–34]) and we show here that FUr also stabilizes p53. Thus, it is conceivable that stabilization of full-length p53 by FUr contributes to the high levels of p53 in R213X nonsense mutant *TP53* cells upon treatment with FUr. However, our previous finding that the Mdm2 inhibitor Nutlin-3a, which inhibits proteasomal degradation of p53 and therefore causes robust p53 stabilization, did not induce significant levels of full-length p53 in neither HDQ-P1 nor H1299-R213X cells [12], demonstrates that stabilization of low levels of full-length p53 produced by basal translational readthrough is by itself not sufficient to generate substantial levels of full-length p53 in cells carrying endogenous or exogenous R213X mutant *TP53*.

FUr is incorporated into RNA [26], and we show here that FUr is to a large extent substituted for uridine, especially in mRNA (Fig. 6B). This efficient incorporation of FUr is most likely the reason for the time-dependent decrease in free intracellular FUr levels (Table S1). Stop codon readthrough by 5-FU has previously been reported in dual-luciferase reporter plasmids and suggested to be due to inhibition of rRNA pseudouridylation [39]. We also show that combination treatment with FUr and uridine causes a dose-dependent decrease in full-length p53 levels. Based on these results, we postulate that one mechanism by which FUr induces translational readthrough is incorporation into mRNA. This is similar to the recently discovered compound clitocine, which works by adenosine replacement but unfortunately is highly toxic [17, 40], and in contrast to aminoglycosides that appear to induce readthrough via interactions with the ribosome [23].

Interestingly, studies in *E. coli* have shown that 5-FU incorporated into mRNA can base-pair with guanine [41, 42]. The phenotype of one *E. coli* nonsense mutant could be reversed by 5-FU as result of mispairing as cytosine in the translation process [42]. This suggests that the presence of FUr instead of uridine at the R213X UGA PTC would make the PTC resemble a CGA codon and thus allow base-pairing with Arg tRNA and translation of full-length p53 with a WT Arg residue at codon 213. This might lead to a complete restoration of WT p53 expression, which would account for the observed transcriptional transactivation activity of FUr-induced p53.

Our p53 DNA binding and p53 reporter assays clearly demonstrate that the full-length p53 induced by FUr is functional as transcription factor. This is also confirmed by the upregulation of p53 target genes. Although FUr and G418 induced translational readthrough of R213X mutant *TP53* with comparable efficiency, it is interesting to note that their p53 target gene activation profiles were different. The stronger upregulation of target genes related to apoptosis, i.e. Fas (extrinsic apoptosis pathway) and Puma (intrinsic apoptosis pathway) by FUr as compared to G418 in H1299-R213X cells correlated with a higher fraction of Annexin V-positive cells after treatment with FUr compared to G418. Puma was also induced more potently by FUr compared to G418 in HDQ-P1 cells. This indicates that even though less full-length p53 protein is produced after FUr treatment compared to G418, FUr-induced full-length p53 is a more potent apoptosis inducer than full-length p53 induced by G418. This difference could be related to post-translational p53 modifications in response to FUr-induced cellular stress, as well as additional pro-apoptotic effects of FUr. It is also possible that translational readthrough induced by G418 has a lower propensity for insertion of the WT Arg residue at codon 213 as compared to FUr, and therefore produces less amounts of active p53 protein.

FUr was also capable of inducing full-length p53 in cells carrying two other *TP53* nonsense mutations, E349X and Q192X, with a potency even higher than that for R213X. The Q192X and E349X mutations give rise to UAG and UAA PTCs, respectively, both of which show a lower propensity for translational readthrough as compared to UGA [43]. However, both contain a cytosine as the fourth nucleotide (+4), if counting the first nucleotide in the PTC as +1, which has been reported to favor readthrough induction [25, 44, 45]. Although the R196X mutation gives rise to a UGA, it showed the lowest level of readthrough after treatment with FUr among the tested *TP53* PTCs. This could possibly be explained by the fact that the R196X PTC has a guanine as the +4 nucleotide whereas R213X which also gives rise to a UGA has a cytosine as the +4 nucleotide. It is noteworthy that efficient readthrough induced by gentamicin was associated with a uracil at the -1 nucleotide position and cytosine at the +4 position [45] and that the same nucleotides are found in *TP53* Q192X and R213X.

As discussed above, 5-FU and FUr can stabilize and activate p53 as part of a cellular stress response. Therefore, our results indicate a unique dual mechanism of action of FUr on tumor cells that carry nonsense mutant *TP53*. First, incorporation of FUr at the R213X PTC in *TP53* mRNA allows translational readthrough of full-length p53 protein which is fully functional, as discussed above. Second, FUr-induced cellular stress could lead to further stabilization and activation of full-length p53 by post-translational modifications, and subsequently upregulation of p53 target genes and potent apoptotic cell death.

In conclusion, we present a novel mechanism for the anticancer agent 5-FU as a readthrough inducer of nonsense mutant *TP53* through its metabolite FUr. This is of particular interest given that p53 is crucial for the apoptotic response of tumor cells to 5-FU [34]. Thus, 5-FU/FUr not only restores expression of full-length p53 in tumor cells that carry nonsense mutant *TP53* but also triggers cell death by apoptosis via p53. This has clinical implications. First, *TP53* nonsense mutation may increase the sensitivity of a tumor to 5-FU, suggesting that information about *TP53* mutation status should guide the selection of therapeutic agent. Second, FUr itself should be explored as therapeutic agent in patients with nonsense mutant *TP53* tumors. This might enable rescue of full-length functional p53 and reduce the side effects of 5-FU that are associated with DNA damage induced by FdUr. Third, the translational readthrough activity of FUr could also be relevant for nonsense mutations in other cancer-related genes, for instance *APC*, *PTEN*, *RB1* and *TET2*. The effect of FUr on nonsense mutations in these genes deserves further investigation.

## Materials and Methods

### NCI-60 database data analysis

The Developmental Therapeutics Program (DTP, http://dtp.cancer.gov/) at the National Cancer Institute (NCI) provides data of a large number of low molecular weight compounds in a panel of 60 human tumor cell lines representing different tumor types [46, 47]. We focused our analysis on colon and renal tumors as these were the only tumor types with *TP53* nonsense mutations in the database. Cell lines from both tumor types were divided by *TP53* status: nonsense mutant *TP53*-carrying tumors, wild-type (WT) *TP53*-carrying tumors, and tumors carrying missense or other types of *TP53* mutations. For colon cancer cell lines, the only cell line with *TP53* nonsense mutation was HCC-2998. HCT116 carries WT *TP53* while HCT-15, HT29, KM12, SW-620 and COLO205 carry missense mutant *TP53*. Among the renal cancer cell lines, SN12C has nonsense mutant *TP53* whereas A498, ACHN, CAKI-1 and UO-31 carry WT *TP53* and RXF-393, 786-0 and TK-10 harbor missense mutant *TP53*. We extracted the mean 50% growth-inhibitory concentrations (GI_50_) for 47 000 compounds in the 15 cell lines and selected compounds that fulfilled two requirements in both cancer types: 1) mean GI_50_ of tumor cells carrying other *TP53*-mutations/mean GI_50_ of nonsense mutant *TP53*-carrying tumor cells >4, and 2) mean GI_50_ of WT *TP53*-carrying tumor cells/mean GI_50_ of nonsense mutant *TP53*-carrying tumor cells >1.5. Schematic representation of the analysis is provided in Fig. S1A and S1B. Data used for the analysis were downloaded from http://dtp.cancer.gov/.

### Cells and cell culture

HDQ-P1 human breast carcinoma cells carrying endogenous R213X nonsense mutant *TP53* (DSMZ, Braunschweig, Germany) were grown in DMEM low glucose medium (Hyclone, USA) supplemented with 10% fetal bovine serum (FBS) (Gibco, USA). H1299 human lung adenocarcinoma parental cells (ATCC, USA), which are p53 null because of a homozygous multi-exon deletion that disrupts the coding sequence of *TP53*, were stably transfected with R213X nonsense mutant *TP53* or other plasmid constructs as described [12]. Stably transfected cells were cultured in RPMI-1640 medium (Hyclone, USA) supplemented with 25 mM HEPES Buffer solution (Gibco, USA) and 10% FBS (Gibco, USA). Detailed information about the constructs used to generate the different H1299 sublines is provided in Table S2. All plasmid constructs were made by GenScript (USA).

HCT116 sublines with sfGFP or sfGFP150X readthrough reporter were generated by piggy-bac-mediated transposition [48]. Briefly, parental cells were seeded the day before transfection and cotransfected with the reporter and piggy-bac transposase plasmids in a 4:1 ratio. Earliest 48 h after transfection, cells were split and selected with blasticidin (200 – 1500 μg/ml) for 7d. Stable integrant cell lines were recovered in DMEM (DMEM, GlutaMAX, Thermo Fisher Scientific, USA) and 10% FBS (Sigma-Aldrich/Merck, Germany). HCT116 parental cells (a kind gift from Bert Vogelstein, Johns Hopkins Oncology Center, Baltimore) and stably transfected HCT116 cells were grown in McCoy′s 5A medium (Sigma-Aldrich/Merck, Germany) supplemented with 10% FBS (Gibco, USA).

### Drugs and chemicals

5-Fluorouracil (5-FU; CAS Number: 51-21-8), 5-Fluorouridine (FUr; CAS Number: 316-46-1), 5-Fluoro-2’-deoxyuridine (FdUr; CAS Number: 50-91-9) and uridine (CAS Number: 58-96-8) were obtained from Sigma-Aldrich/Merck (Germany) and dissolved in water, except 5-FU which was dissolved in DMSO (Sigma-Aldrich/Merck, Germany). Geneticin™ Selective Antibiotic (G418 Sulfate) (50 mg/ml) which is supplied as a solution in water was obtained from Gibco (USA). For animal studies, both 5-FU and FUr were diluted in vehicle with a final concentration of 10% DMSO in DPBS (Dulbecco’s Phosphate Buffered Saline).

### Human tumor xenografts and in vivo treatments

All animal studies were approved by the Stockholm Animal Experiments Ethical Committee, Sweden (Dnr 7054-2019; Dnr 15763-2020). Animal care was in accordance with Karolinska Institutet guidelines: experimental mice were housed in enriched individually ventilated cages in a 12-hour light/dark cycle at 19–23 °C and 50–65% air humidity at a pathogen-free animal facility at Karolinska Institutet (Stockholm, Sweden). The mice were fed irradiated and CRM(P) mouse pellets (SDS Diets, Essex, UK) and drinking water *ad libitum*. Twelve female athymic nude mice (Crl:NU(NCr)-*Foxn1*^*nu*^) were purchased from Charles River (Sulzfeld, Germany). At 6 weeks of age, all mice were subcutaneously inoculated with 2 × 10^6^ H1299-R213X-FLAG cells into the right flank and 2 × 10^6^ H1299-R213X-ΔC-FLAG cells into the left flank, both cell lines in 50% BD Matrigel Matrix High Concentration, Phenol Red-Free (354262) from Corning, USA. Until treatment start, mice were weighed once per week and tumor growth was assessed twice per week using a digital caliper. The tumor volume was calculated using the formula (length × width^2^)/2. When the tumor volume in either flank reached >300 mm^3^, treatment was initiated using either 5-FU (50 mg/kg in 10% DMSO, *n* = 4), FUr (10 mg/kg in 10% DMSO, *n* = 4) or vehicle (10% DMSO, *n* = 4). Treatments were made by intraperitoneal injection (IP) daily for 5 days. During the treatment period, mice were weighed daily and tumor volume was measured 3-4 times. Maximum allowed weight loss was 20% and maximum tumor volume 1000 mm^3^ per flank. Mice were sacrificed 1-2 hours after the last injection. Tumors were dissected, weighed and cut in half: one half was frozen in liquid nitrogen for protein analysis, and one half was fixed in 4% formaldehyde for histological and immunohistochemical analysis.

### Western blotting

For experiments in cultured cells, 3 × 10^5^ HDQ-P1 cells per well or 1.5 × 10^5^ H1299-R213X, H1299-R213X-EGFP, H1299-R213X-ΔC-EGFP, H1299-EGFP-X-FLAG, H1299-R213X-FLAG, HCT116 parental, HCT116 sfGFP or HCT116 sfGFP150X cells per well were seeded in 6-well plates in a final volume of 2 ml. On the following day, cells were treated with different concentrations of the indicated drugs for 24, 48 or 72 h. Both attached and floating cells were then harvested using Trypsin-EDTA (Sigma-Aldrich/Merck, Germany) and washed with DPBS (Dulbecco’s Phosphate Buffered Saline) (Sigma-Aldrich/Merck, Germany). Cells were lysed with lysis buffer containing 100 mM Tris pH 7.4, 150 mM NaCl and 1% NP-40. Protease Inhibitor Cocktail (Sigma-Aldrich/Merck, Germany) was added prior to use.

Collected xenograft tumors were flash-frozen in liquid nitrogen. Tumors were homogenized in tubes pre-filled with ceramic beads (Precellys Lysing Kit, Tissue homogenizing CKMix from Bertin Technologies, France) and ice-cold RIPA lysis buffer (150 mM NaCl, 1% NP-40, 0.5% sodium deoxycholate, 0.1% SDS, 50 mM Tris-Cl pH 8 and 5 mM EDTA) with Protease Inhibitor Cocktail (Sigma-Aldrich/Merck, Germany) and Halt™ Phosphatase Inhibitor Cocktail (Thermo Scientific, USA) added prior to use, using a Minilys personal homogenizer (Bertin Technologies, France). Samples were then lysed on ice for 30 min and lysates were cleared twice by centrifugation (13 000 RPM for 10 min at 4 °C).

Protein was quantified using Bradford protein assay (Bio-Rad, USA) or DC™ Protein assay (Bio-rad, USA) and absorbance was measured using SmartSpec™ Plus Spectrophotometer (Bio-Rad, USA) or Varioskan™ LUX multimode microplate reader (Thermo Scientific, USA). After boiling the protein samples in NuPAGE^™^ LDS Sample Buffer and NuPAGE™ Sample Reducing Agent (both from Thermo Fisher Scientific, USA), they were loaded into NuPAGE™ 10% Bis-Tris gels and run in NuPAGE™ MOPS SDS Running buffer (both from Thermo Fisher Scientific, USA). Proteins were transferred to nitrocellulose membranes using iBlot™ 2 Gel Transfer Device (Thermo Fisher Scientific, USA). Membranes were blocked with 5% milk in DPBS containing 0.1% Tween-20 (DPBS-T) for 1 h at room temperature and blotted overnight at 4 °C or 1-2 h at room temperature with HRP-conjugated antibodies from Santa Cruz (USA) with a 1/5 000 to 1/10 000 dilution of anti-p53 antibody DO-1 (sc-126), a 1/1 000 dilution of anti-p21 antibody F-5 (sc-6246), a 1/1 000 to 1/10 000 dilution of anti-GFP antibody B-2 (sc-9996), or a 1/20 000 to 1/30 000 dilution of anti-GAPDH antibody 0411 (sc-47724) or with HRP-conjugated anti-FLAG M2 antibody A8592 (Sigma-Aldrich/Merck, Germany) used at 1/10 000 dilution. Truncated GFP was detected with a 1/2 000 dilution of anti-GFP primary antibody (#ab6556) from Abcam (UK) and cleaved PARP was detected with the primary antibody D214 against Cleaved PARP (#9541, Cell Signaling, USA) diluted 1/1 000. To detect both proteins, the HRP-conjugated secondary antibody anti-rabbit IgG (65-6120, Invitrogen, USA) diluted 1/5 000 was used. Proteins were then visualized with SuperSignal™ West Femto Maximum Sensitivity Substrate (Thermo Scientific, USA) in a FUJIFILM LAS-1000 Image Analyzer (Fujifilm, Japan) or in an iBright FL1000 Imaging System (Thermo Fisher Scientific, USA). Western blotting results for H1299-R213X-FLAG xenograft tumors were quantified with ImageJ (Fiji) software and expression of the protein of interest was normalized to GAPDH expression as loading control. All the original Western blots are provided in Supplemental material – Original Western blots.

### Immunohistochemistry (IHC) and histology

Collected xenograft tumors were fixed in formaldehyde solution 4%, buffered, pH 6.9 (Sigma-Aldrich/Merck, Germany) overnight at room temperature and then transferred to 70% ethanol. Fixed tumors were processed in the automated tissue processing machine LOGOS (Milestone Medical, Italy) and paraffin-embedded. Tumor sections of 4 µm thickness were collected on Superfrost™ Plus Adhesion Microscope Slides (Epredia, USA) and heated for 3 h at 56 °C. Slides were deparaffinized in xylene and rehydrated through a descending ethanol series. Heat-induced epitope retrieval was performed using a Decloaking Chamber™ (Biocare Medical, USA) set at 110 °C for 5 min in EDTA buffer pH 8.5 (E1161 Sigma-Aldrich/Merck, Germany). For quenching of endogenous peroxidase, slides were incubated for 30 min in 0.15% hydrogen peroxidase at room temperature followed by a blocking step using Background Sniper Blocking Reagent (Biocare Medical, USA) for 15 min. Tumor sections were incubated overnight at 4 °C in a humid chamber with HRP-conjugated anti-FLAG M2 antibody A8592 (Sigma-Aldrich/Merck, Germany) diluted 1/500 in Renoir Red Diluent (Biocare Medical, USA). For protein detection the MACH 1 Universal HRP-Polymer Detection Kit was used (Biocare Medical, USA) according to the manufacturer’s protocol. The sections were counterstained in Mayer’s hematoxylin for 1 min followed by dehydration with graded ethanol, xylene and cover slipped using Pertex® Mounting Medium (HistoLab, Sweden).

For histological analysis using Hematoxylin and Eosin (H&E) staining, slides were stained for 5 min with Mayer’s hematoxylin and 45 sec in 0.2% eosin (HistoLab, Sweden), dehydrated in increasing ethanol, xylene for 2 min twice, and finally cover slipped using Pertex® Mounting Medium.

Stained slides were analyzed using Quantitative Pathology & Bioimage Analysis QuPath [49] available at https://qupath.github.io/. Two sections of the same tumor were analyzed. Total tumor area and FLAG-positive area was determined using *Pixel classification*. These areas were used to determine percentage of positive FLAG area staining for each tumor section. Data is presented as mean ± standard error of the mean (SEM) of the two sections of the same tumor and the four tumors in each treatment group.

### Quantitative real-time PCR (qRT-PCR)

1.5 × 10^5^ HDQ-P1 cells per well and 1.5 × 10^5^ H1299-R213X cells or H1299-EV cells per well were seeded in 6-well plates in a final volume of 2 ml. On the following day, cells were treated with different concentrations of the indicated drugs for 72 h. Both attached and floating cells were then harvested using Trypsin-EDTA (Sigma-Aldrich/Merck, Germany) and washed with DPBS (Sigma-Aldrich/Merck, Germany). RNA extraction was performed using the RNeasy mini kit (Qiagen, Germany) according to the manufacturer’s recommendations. RNA was quantified using a NanoDrop Spectrophotometer (Thermo Scientific, USA). cDNA was synthesized from RNA using the SuperScript II Reverse Transcriptase (Thermo Fisher Scientific, USA). Real-time quantitative PCR was performed in the QuantStudio™ 7 Flex Real-Time PCR System (Applied Biosystems, USA) using TaqMan Gene Expression Assays and FastStart Universal Probe Master (Rox) (Roche, Switzerland). TaqMan probes used were *TP53* (Hs00153340_m1), *ZMAT3* (Hs00536976_m1), *CDKN1A* (Hs00355782_m1), *FAS* (Hs00531110_m1), *BAX* (Hs00414514_m1), *PMAIP1* (Hs00560402), *BBC3* (Hs00248075_m1) and *GAPDH* (Hs99999905_m1) (Applied Biosystems, USA), corresponding to p53, Zmat3 (Wig-1), p21, Fas, Bax, Noxa, Puma and GAPDH, respectively. Relative gene expression was calculated by the 2–ΔΔCt method using GAPDH as endogenous control.

### Flow cytometry

To determine readthrough induction by EGFP reporter gene expression, H1299-R213X-EGFP and H1299-EV cells were seeded at a density of 0.75 × 10^5^ cells/well in 12-well plates in a final volume of 1 ml. On the following day, cells were treated with different concentrations of G418, 5-FU, FUr, FdUr or uridine. Depending on the vehicles of the compounds used in each experiment, cells were left untreated or were treated with DMSO as negative control. After 72 h incubation, attached and floating cells were harvested using Trypsin-EDTA (Sigma-Aldrich/Merck, Germany) and washed with DPBS. Cells were resuspended in 150 µl of DPBS and analyzed with NovoCyte flow cytometer (ACEA Biosciences, San Diego, California, USA). Collected events were first gated using side scatter-height (SSC-H)/forward scatter-height (FSC-H) and single cells were gated using forward scatter-height (FSC-H)/forward scatter-area (FSC-A) gates. From those selected events, cells were determined as EGFP positive or negative by assessing EGFP signal. Data was analyzed by subtracting the percentage of EGFP positive H1299-EV cells from EGFP positive H1299-R213X-EGFP cells.

To examine p53 activity with an EGFP reporter construct, H1299-R213X and H1299-EV cells were seeded at a density of 1.5 × 10^5^ cells/well in 6-well plates in a final volume of 2 ml. On the next day, cells were transfected with 1 µg of a DNA construct carrying 13 consensus p53 DNA binding motifs followed by EGFP using Lipofectamine™ 2000 Transfection Reagent (Thermo Fisher Scientific, USA) according to the manufacturer’s recommendations. After 24 h, media was changed to remove excess of plasmid and cells were treated with FUr, FdUr or G418 or left untreated as negative control for 72 h. Attached and floating cells were then harvested using Trypsin-EDTA (Sigma-Aldrich/Merck, Germany) and washed with DPBS. Cells were resuspended in 150 µl of DPBS and analyzed with NovoCyte flow cytometer (ACEA Biosciences, San Diego, California, USA). Collected events were first gated using SSC-H/FSC-H and single cells were gated using FSC-H/FSC-A gates. From those selected events, cells were determined as EGFP positive or negative by assessing EGFP signal. Data was analyzed by subtracting the percentage of EGFP positive H1299-EV cells from EGFP positive H1299-R213X cells.

To assess cell death by Annexin V, H1299-R213X-EGFP, H1299-R213X-ΔC-EGFP, H1299-EV, H1299 parental, H1299-R213X-ΔC-Flag, H1299-R213X and HDQ-P1 cells were seeded at a density of 1.5 × 10^5^ cells/well in 6-well plates in a final volume of 2 ml. Next day, cells were treated with different concentrations of FUr, FdUr or G418 or left untreated as negative control. After 72 h incubation, attached and floating cells were harvested using Trypsin-EDTA (Sigma-Aldrich/Merck, Germany) and resuspended in DPBS containing calcium chloride and magnesium chloride (Gibco, USA). Cells were counted using Countess II Automated Cell Counter (Thermo Fisher Scientific, USA) and 1 × 10^5^ cells per condition were aliquoted. Cells were spun down and resuspended in 100 μl of Annexin V binding buffer 1X and incubated with 4 μl of BD Horizon™ V450 Annexin V antibody (BD Biosciences, USA) or with 5 µl of Annexin V Alexa Fluor™ 647 conjugate (A23204, Thermo Fisher Scientific, USA) for 15 minutes in the dark at room temperature. Cells were spun down at 3 000 RPM for 10 minutes and washed with ice-cold DPBS containing calcium chloride and magnesium chloride. Cells were resuspended in 150 μl DPBS containing calcium chloride and magnesium chloride and analyzed with NovoCyte flow cytometer (ACEA Biosciences, USA). Collected events were first gated using SSC-H/FSC-H and single cells were gated using FSC-H/FSC-A gates. From these selected events, cells were classified as Annexin V positive or negative, and EGFP positive or negative in case of experiments with H1299-R213X-EGFP and H1299-R213X-ΔC-EGFP cells. All flow cytometry data was analyzed using the NovoExpress Software (Agilent, USA).

### Enzyme-Linked Immunosorbent Assay (ELISA)

H1299-R213X-ΔC-FLAG cells were seeded at a density of 0.5 × 10^6^ cells per 5 cm dish in a final volume of 5 ml. On the following day, cells were treated with 5-FU, FUr, FdUr or G418 for 72 h, and then harvested and lysed in lysis buffer containing 100 mM Tris pH 7.4, 150 mM NaCl and 1% NP-40. Protease Inhibitor Cocktail (Sigma-Aldrich/Merck, Germany) was added prior to use. ELISA plates were coated overnight at 4 °C with 0.5 µg FLAG M2 antibody (F1804, Sigma-Aldrich/Merck, Germany) in 100 µl of 0.1 M carbonate buffer pH 9.2 per well. Plates were then blocked with 200 µl blocking buffer (5% milk in DPBS-T) for 2 h at 4 °C and washed three times with DPBS-T. 100 µg protein in 50 µl lysis buffer and 50 µl blocking buffer were added to the wells and plates were incubated overnight at 4 °C. Plates were washed three times with DPBS-T. 100 µl of 1/500 diluted p53 DO-1 HRP-conjugated antibody (sc-126, Santa Cruz, USA) were added to each well and incubated for 2 h at 4 °C and plates were washed three times with DPBS-T. ELISA was developed by adding 100 µl of 1-Step™ Ultra TMB-ELISA Substrate Solution (34028, Thermo Fisher Scientific, USA) and incubation at room temperature. Once adequate signal was obtained, reaction was stopped by adding 10 µl of 1 M HCl to each well and absorbance was measured at 450 nm with Varioskan™ LUX multimode microplate reader (Thermo Scientific, USA).

### p53 DNA binding assay

1.5 × 10^5^ H1299-R213X cells per well were seeded in 6-well plates in a final volume of 2 ml. Next day, cells were treated with 5 µM FUr or FdUr or 50 µM G418 for 72 h. Both attached and floating cells were then harvested using Trypsin-EDTA (Sigma-Aldrich/Merck, Germany) and washed with DPBS (Sigma-Aldrich/Merck, Germany). The nuclear Extraction Kit (ab113474, Abcam, UK) was used to prepare the nuclear extracts following the manufacturer’s recommendations. p53 DNA binding activity was examined using TransAM® p53 DNA-binding ELISA for activated p53 (Active Motif, Germany) following the manufacturer’s recommendations and using 10 µg of nuclear extract. Absorbance was measured at 450 nm with a Varioskan™ LUX multimode microplate reader (Thermo Scientific, USA).

### RNA-seq and Ribosome profiling (Ribo-seq)

Ribosome profiling was performed as described previously [50, 51], with several modifications. H1299-R213X and H1299-EV cells were seeded at a density of 0.3 × 10^6^ cells/15 cm dish for non-treated and G418-treated conditions or at a density of 2.3 × 10^6^ cells/15 cm dish for FUr-treated condition in a final volume of 32 ml. Cells were seeded at different densities to obtain a sufficient number of cells after each treatment for further sample processing. One day after seeding the cells, they were treated with 100 µM of G418 or 3 µM of FUr or left untreated for 72 h, using four replicate treatments per condition. Next, medium was aspirated, cells were washed once with 5 ml of ice-cold DPBS, and plates were snap frozen with liquid nitrogen. Snap-frozen cells were scraped off in 1 ml of ice-cold lysis buffer (20 mM Tris-Cl pH 7.4 (Sigma-Aldrich, USA), 150 mM NaCl (Fisher Scientific, USA), 5 mM MgCl_2_ (Fisher Scientific, USA), 1% Triton X-100 (Sigma-Aldrich, USA), 0.1% IGEPAL CA-630 (Sigma-Aldrich, USA), 1 mM DTT (Sigma-Aldrich, USA), 10 U/ml RNase-free DNase 1 (Lucigen, USA), 100 µg/ml cycloheximide (Sigma-Aldrich, USA) and transferred to Eppendorf tubes for lysis on ice for 10 min. Lysates were clarified through centrifugation at 20 000 g at 4 °C for 10 min. Total RNA content of sample lysates was estimated using the Qubit™ RNA broad range (BR) Assay Kit (Thermo Fisher Scientific, USA) on a Invitrogen™ Qubit™ 4 fluorometer (Thermo Fisher Scientific, USA). Lysates were then digested per 200 µl aliquots with RNAse I (Lucigen, USA) for generation of ribosome-protected fragments (RPFs), using 20 units of RNAse I per 20 µg of RNA measured in lysate. After 45 min of digestion at 23 °C while shaking at 400 RPM on a thermomixer, the digestion reaction was stopped by adding 10 µl (10U) of SUPERase*In RNAse inhibitor and placing the samples on ice (Thermo Fisher Scientific, USA). Digested lysates were then transferred to Microspin S-400 HR sephacryl columns (Sigma-Aldrich, USA) equilibrated with 3 ml of cold polysome buffer (20 mM Tris-Cl pH 7.4, 150 mM NaCl, 5 mM MgCl_2_) each and centrifuged at 600 g at room temperature for 2 min using an Eppendorf 5425 R microcentrifuge. 20 µl 10% SDS were then added to digested lysates and RPFs were then extracted using 3 V (660 µl) Trizol LS (Fisher Scientific, USA), followed by RNA purification using the Zymo Direct-zol RNA micro prep kit (Zymo Research, USA) with modifications to the manufacturer’s instructions: Columns were spun dry for 2 min at 12 000 g and isolated RPFs were eluted in 20 µl nuclease-free water (Sigma-Aldrich, USA).

rRNA was depleted using the RiboPOOL technology (siTOOLs Biotech; human Ribo-Seq riboPOOL (ID: 42); cat# dp-K012-000042, Germany) with modifications to the manufacturer’s instructions: 200 pmol of RiboPOOL and 100 µl of beads were used per sample. RNA was purified using the Zymo RNA Clean and Concentrator-5 kit (Zymo Research, USA) with modifications to the manufacturer’s instructions as mentioned above. Purified RNA was then size-selected through denaturing PAGE using 15% TBE-Urea gels (Thermo Fisher Scientific, USA). RNA fragments corresponding to 26–34 nucleotides were excised and recovered from gel slices by rocking at 37 °C at 700 RPM on a thermomixer. RNA solutions were transferred to Costar Spin-X filter tubes (Thermo Fisher Scientific, USA) and filtered through centrifugation at 5 000 RPM for 6 min using an Eppendorf 5425 R microcentrifuge. 2 µl of GlycoBlue (Thermo Fisher Scientific, USA) and 700 µl of isopropanol were then added per aliquot and RNA was left to precipitate overnight at −80 °C.

Following precipitation, RNA fragments were pelleted by centrifugation at 21 000 g at 4 °C for 45 min. Pellets were washed once with 800 µl of ice-cold ethanol and centrifuged at 21 000 g at 4 °C for 15 min. RNA pellets were then air-dried for 3-4 min and dissolved in 60.75 µl nuclease-free water on ice. RNA fragments were dephosphorylated using 30 U of T4 PNK (Lucigen, USA) for 1 h at 37 °C and purified using Zymo RNA Clean and Concentrator-5 kit (Zymo Research, USA), where isolated RNA fragments were eluted in 9.5 µl nuclease-free water (Sigma-Aldrich, USA). Purified fragments were then ligated to a pre-adenylated 3’oligonucleotide linker using 100 U of T4 RNA ligase 2 Deletion Mutant (Lucigen, USA) and 5 U T4 RNA ligase 1 (Thermo Fisher Scientific, USA) at 23 °C for 3 h. Leftover linker was removed using 5’Deadenylase (New England Biolabs, USA) and Rec J Exonuclease (Lucigen, USA). Linker-ligated RNA fragments were reverse-transcribed into cDNA using EpiScript reverse transcriptase (Lucigen, USA). cDNA was treated with Exonuclease I (Lucigen, USA) for 30 min at 37 °C, followed by 15 min at 80 °C with a reduction to 4 °C; and further treated with 5 U of RNAse I (Lucigen, USA) and 2.5 U of Hybridase Thermostable RNase H (Lucigen, USA) at 55 °C for 5 min followed by an incubation step at 4 °C to stop the reaction. Treated cDNA was purified using the Zymo Oligo Clean and Concentrator Kit (Zymo Research, USA) with modifications to the manufacturer’s instructions: Columns were spun dry for 2 min at 12 000 g and isolated RPFs were eluted in 9.5 µl nuclease-free water (Sigma-Aldrich, USA).

cDNA containing ligated linkers was size-selected through denaturing PAGE using 10% TBE-Urea gels (Thermo Fisher Scientific, USA). cDNA fragments corresponding to 70–80 nucleotides were excised and precipitated with ammonium acetate and SDS followed by overnight precipitation with isopropanol as described above. Size-selected cDNA was circularised for 3 h at 60 °C using 100 U of circLigase I (Lucigen, USA) followed by heat inactivation for 10 min at 80 °C and amplified using Phusion high-fidelity polymerase (New England Biolabs, USA) with reverse primers containing unique barcode sequences for 10 cycles of: 30 sec at 98 °C, 15 sec at 94 °C, 5 sec at 55 °C, 10 sec at 65 °C. Following amplification, 5 µl of 3 M NaCl (Thermo Fisher Scientific, USA), 1 ml of ethanol and 2 µl of GlycoBlue (Thermo Fisher Scientific, USA) were added to each aliquot of cDNA and left to precipitate overnight at −80 °C.

Amplified cDNA libraries were size-selected using 7% non-denaturing TBE-Urea gels (Thermo Fisher Scientific, USA). cDNA libraries corresponding to 150 nucleotides were excised and recovered from gel slices by rocking at 37 °C at 700 RPM on a thermomixer. cDNA solutions were then transferred to Costar Spin-X filter tubes (Thermo Fisher Scientific, USA) and filtered through centrifugation at 5 000 RPM for 6 min using an Eppendorf 5425 R microcentrifuge. cDNA libraries were purified using the Zymo DNA Clean and Concentrator-5 kit (Zymo Research, USA) with modifications to the manufacturer’s instructions: Columns were spun dry for 2 min at 12 000 g and isolated RPFs were eluted in 13 µl nuclease-free water (Sigma-Aldrich, USA).

cDNA libraries were quantified using Qubit™ DNA high sensitivity (HS) Assay Kit (Thermo Fisher Scientific, USA) according to the manufacturer’s instructions on an Invitrogen™ Qubit™ 4 fluorometer (Thermo Fisher Scientific, USA) and Bioanalyzer 2100 (Agilent) using the High Sensitivity DNA kit and pooled in equimolar ratios. Sequencing was performed on a NextSeq2000 (Illumina; 1x50bp) at the Utrecht Sequencing Facility (USEQ) to a depth varying between 21 009 133 and 43 370 799 total reads per sample.

For RNA sequencing, total RNA was isolated from 200 µl of lysate using 750 µl of TRIzol LS (Thermo Fisher Scientific, USA) and purified using the Zymo RNA Clean and Concentrator-5 kit (Zymo Research, USA) with modifications to the manufacturer’s instructions: Recovered RNA was treated with DNAse I (Lucigen, USA) at 37 °C for 10 min prior to addition of RNA Binding Buffer, and isolated RNA was eluted in 15 µl nuclease-free water (Sigma-Aldrich, USA). Prior to library preparation, purified RNA was quantified and checked for RNA integrity (RIN values 8,7 – 10) with Bioanalyzer 2100 (Agilent) using the RNA 6000 Nano kit according to the manufacturer’s instructions. With 1.5 µg of total RNA as input, mRNA-seq libraries were prepared using TruSeq Stranded mRNA library prep kit (Illumina), multiplexed, and sequenced in a 2x150bp paired-end fashion on a NovaSeq™ 6000 (Illumina) at GENEWIZ (Leipzig, Germany) to a depth varying between 49 489 998 and 104 879 962 total reads per sample.

### RNA-seq and Ribo-seq data processing and mapping

Prior to mapping, ribosome-profiling adapters were clipped from reads and reads were filtered for the standard quality threshold used by CutAdapt v3.4 (ref. [52]). Fragments shorter than 25 nucleotides were discarded. Next, reads were mapped to mitochondrial, ribosomal, small nuclear, small nucleolar, and transfer RNA sequences using Bowtie2 v2.4.2 (ref. [53]), as described previously. All reads not mapped to this custom annotation were kept and mapped with STAR v2.7.8a to the human genome, guided by a reference transcriptome annotation (Ensembl human gene annotation release 102). A maximum of two mismatches (*--outFilterMismatchNmax*) and 20 multimapping sites (*--outFilterMultimapNmax*) were allowed. We used RiboseQC v1.1 (ref. [54]) with the *max_inframe* setting to check the quality of each Ribo-seq sample and to extract sequence information, dominant read lengths (footprint distributions), periodicity (3nt codon movement), and P site counts from uniquely mapped reads.

mRNA-seq reads were first evaluated with CutAdapt v3.4 for standard QC metrics. We used STAR v2.7.8a guided with a reference annotation (Ensembl human gene annotation release 102) to map the qualifying reads. A maximum of six mismatches *(--outFilterMismatchNmax*) and 10 multimapping sites (*--outFilterMultimapNmax*) were allowed. In addition, the STAR mapping score normalised on read length should at least be higher than 0.75 (*--outFilterScoreMinOverLread*). To integrate and jointly normalize and analyze mRNA-seq and Ribo-seq data, we further performed all mRNA-seq quantifications using CDS-mapped, single-end reads trimmed to match ribosomal footprint sizes, which were processed and mapped exactly according to the Ribo-seq data as described [51].

### Gene quantification and differential expression analysis

To quantify gene expression levels, we used FeatureCounts from the subread package v2.0.2 (ref. [55]) to count sequenced reads mapping to coding sequence (CDS) regions of genes. We used DESeq2 v1.30.1 (ref. [56]) to normalize read counts, estimate size factors and perform differential expression analysis. Pairwise Pearson correlations were calculated using variance-stabilizing transformed matrices of the full gene list.

### Translational readthrough quantification

To measure readthrough inducing treatment effects, we looked at *TP53*-specific premature stop codon readthrough, as well as readthrough on translatome-wide level. We used normalized CDS coverage as provided by the RiboseQC pipeline [54] to visualize and quantify premature stop (R213X) readthrough of ectopically expressed *TP53* upon G418, FUr, or control treatments. Readthrough ratios were calculated using the following formula: (R213X_post_ CDS coverage)/(R213X_pre_ CDS coverage), using coverage values based on the total number of counted reads, normalized by length of the evaluated *TP53* coding sequence region (pre or post R213X). To determine readthrough at canonical stop codons at the ends of annotated coding sequences in a translatome-wide fashion, we binned in-frame read coverages generated by RiboseQC [54] and used the meta CDS position at which a strong peak of footprints (which resembles piled-up ribosomes prior to being released from the coding sequence) demarcates the stop codon position in a manner reproducible across all sequenced samples. The percentage of reads assigned to each bin was calculated using the sum of all bins per sample. The readthrough ratio was calculated by taking the binned fraction of in-frame reads before and after the end of the reading frame (i.e., into the annotated 3’ UTR).

### Cell confluence analysis

H1299-R213X, H1299-R213X-EGFP and H1299-EV cells were seeded at a density of 0.75 × 10^5^ cells/well in 12-well plates in a final volume of 1 ml or at a density of 0.3 × 10^4^ cells/well in 96-well plates in final volume of 100 µl. Next day, cells were treated with different concentrations of G418, FUr, FdUr or uridine, or left untreated as negative control. Plates were then placed inside Incucyte® S3 Live-Cell Analysis System (Essen BioScience, USA). Incucyte® S3 Live-Cell Analysis System was programmed to take 4 pictures/well every 3 h using phase channel. After 72 h of image collection, data was analyzed using Incucyte® S3 Analysis Software. Cell confluence data related to Caspase 3/7 activity assay is presented as fold change to time point 0 h. Cell confluence curves related to competition experiments of FUr, FdUr or G418 with uridine were analyzed using an application available at https://vladjnbykov.shinyapps.io/IncuCyte_confluency/ (created by V.J.N. Bykov) based on a logistic regression model. Data from this analysis is presented as cell growth rate for each condition at the time when control population growth rate reaches its maximum or critical point (cp) and is expressed as percentage confluency/h.

### WST-1 assay and IC_50_ calculations

H1299-R213X and H1299-EV cells were seeded at a density of 0.3 × 10^4^ cells per well in 96-well plates in 100 µl. Cells were treated on the following day with different concentrations of 5-FU, FUr, FdUr or G418. Depending on the vehicles of the compounds used in each experiment, cells were left untreated or were treated with DMSO as negative control. After 72 h, cell proliferation reagent WST-1 (Roche, Switzerland) was added according to manufacturer’s protocol and absorbance was determined at 450 nm with Varioskan™ LUX multimode microplate reader (Thermo Scientific, USA). IC_50_ values were calculated with Nonlinear Regression analysis using the Dose-response – Inhibition built-in equation in GraphPad Prism version 9.2.0 (GraphPad Software, USA).

### Caspase 3/7 activity assay

H1299-R213X and H1299-EV cells were seeded at a density of 0.3 × 10^4^ cells/well in 96-well plates in final volume of 100 µl. Next day, cells were treated with different concentrations of FUr or FdUr, or left untreated as negative control, with 3 µM CellEvent Caspase-3/7 Green Detection Reagent (Thermo Fisher Scientific, Stockholm, Sweden) and plates were placed inside Incucyte® S3 Live-Cell Analysis System (Essen BioScience, USA). Incucyte® S3 Live-Cell Analysis System was programmed to take 4 pictures/well every 3 h using phase and green channel for 72 h. Data was analyzed using Incucyte® S3 Analysis Software. The green counts signal obtained from caspase 3/7 activity for each sample was normalized to its cell confluence value obtained with phase channel and further normalized to time point 0 h.

### Liquid chromatography-mass spectrometry (LC-MS) analysis

To assess cellular uptake and stability of compounds in the media, H1299-R213X cells were seeded in 6-well plates at a density of 1.5 × 10^5^ cells/well in a final volume of 2 ml. On the following day, cells were treated with different concentrations of G418, 5-FU, FUr or FdUr, or left untreated as negative control. Just after treatment, media samples for all compounds were collected, corresponding to time point 0 h after treatment. After 24, 48 and 72 h of incubation, media samples for each condition and cells treated with each compound were collected. Both attached and floating cells were harvested using Trypsin-EDTA (Sigma-Aldrich/Merck, Germany) and washed with DPBS (Sigma-Aldrich, USA). Cells were lysed with 20% DPBS and 3 freezing-thawing cycles. Lysates were centrifuged at 14 000 RPM for 15 min at 4 °C and the supernatant was collected. Protein precipitation was performed in both media and cells samples by adding 4 times sample volume of acetone than the initial media or cell lysate volumes and kept overnight at −20 °C. The next day, cells were centrifuged at 14 000 RPM for 15 min at 4 °C and the supernatant was collected. This step was performed twice. All samples were concentrated to almost dryness in SpeedVac (Savant™, Thermo Fisher Scientific, USA) and stored at −20 °C. Samples were reconstituted with 0.1% of formic acid in water and analyzed by LC-MS.

Samples were analyzed on a Waters Alliance HPLC system operated with MassLynx software (Waters, Sweden), equipped with 2998 Photodiode array detector (Waters, Sweden) and ACQUITY QDa Performance MS detector (Waters, Sweden) employing the electrospray ionization technique. A positive ion mode was used for detection and quantification analysis. A LUNA C18(2), 250 × 3 mm, 3 μm particle size, Phenomenex (Værløse, Denmark) column equipped with precolumn and precolumn filter, maintained at 35 °C, was used for the analysis. The injection volume was 10 µl. Separation was performed using gradient elution with water that was mixed with acetonitrile. Formic acid was added to all solvents to a final concentration of 0.1%. The initial elution was isocratic with 1% acetonitrile for 5 min, followed by a linear acetonitrile gradient for 5 min to 10% acetonitrile and then 10 min to 99% acetonitrile. The flow rate was 0.39 ml/min.

To examine incorporation into RNA, H1299-R213X cells were seeded in T175 flasks at a density of 2.4 × 10^6^ cells/flask in a final volume of 32 ml. The day after, cells were treated with different concentrations of G418, 5-FU, FUr or FdUr. After 72 h incubation, both attached and floating cells were harvested using Trypsin-EDTA (Sigma-Aldrich/Merck, Germany) and washed with DPBS (Sigma-Aldrich, USA). RNA extraction was performed with the RNeasy MidiKit kit (Qiagen, Germany) according to the manufacturer’s recommendations. RNA was quantified by a NanoDrop Spectrophotometer (Thermo Scientific, USA) and 5 μg of RNA from each sample were aliquoted as samples corresponding to total RNA longer than 200 bases. The remaining RNA was used for mRNA purification using the Oligotex mRNA kit (Qiagen, Germany) according to the manufacturer’s recommendations. RNA was first pre-digested with 1 U of Benzonase nuclease per sample in the presence of 1 mM magnesium chloride at pH 7.5 for 1 h at 37 °C. Ribonucleotides were then digested with 0.25 U of Nuclease P1 in 20 mM sodium acetate, 0.2 mM zinc chloride pH 5.3 for 2 h at 50 °C. Dephosphorylation was performed with 5 U of Shrimp alkaline phosphatase in the buffer provided by manufacturer for 2 h at 37 °C at pH 8.0. Afterwards, 4 times the sample volume of −20 °C acetone was added and kept for 2 h or overnight at −20 °C. Samples were centrifuged at 14 000 RPM for 15 min at 4 °C and supernatants transferred to a clean tube. This step was repeated twice. Samples were then evaporated not to complete dryness in a SpeedVac (Savant™, Thermo Fisher Scientific, USA) and stored at −20 °C. Finally, samples were diluted in water with 0.1% formic acid and analyzed by LC-MS as described above.

### Data presentation and statistical analysis

Statistical analyses were performed using GraphPad Prism version 9.2.0 (GraphPad Software, USA). Normality of the data was tested with Shapiro-Wilk test. If the data distribution was normal and two groups were compared, two-tailed independent *t*-test was applied; if the distribution was normal but more than two groups were compared, repeated measures one-way ANOVA was applied with either Tukey’s multiple comparisons follow-up test if all groups were compared to each other, or with Dunnett’s multiple comparisons follow-up test if all groups were compared to only the control sample. If the data distribution was not normal and more than two groups were compared, the non-parametric Friedman test was applied with Dunn’s multiple comparisons follow-up test. *p*-values < 0.05 were considered statistically significant. Statistical significance is shown with “*” when a parametric test was applied and with “^#^” when a non-parametric test was applied. Data are presented as mean ± standard error of the mean (SEM) or median ± 95% confidence interval. Image processing was performed using Adobe Photoshop 2021 (Adobe, USA) only when needed for improved visualization. The same processing was applied to the whole image.

## Supplementary information

Supplementary information

Supplemental material - Original Western blots

Reproducibility checklist

## Data Availability

Data obtained with LC-MS on G418, 5-FU, FUr and FdUr cellular uptake, stability in the media, interconversion between 5-FU, FUr and FdUr, and data of effects on different cellular metabolites can also be accessed at https://cellmet.herokuapp.com/. The RNA-seq and Ribo-seq datasets generated and analyzed during the current study are available via NCBI’s Gene Expression Omnibus data repository under GEO accession number GSE196712. RNA-seq and Ribo-seq data analysis was performed using custom software written in R. Analysis scripts and a copy of the pipeline are available at https://bitbucket.org/vanHeeschLab/fur_manuscript/src/master/.
