# Supplementary material for: Translational readthrough of nonsense mutant TP53 by mRNA incorporation of 5-Fluorouridine
Source: Cell Death Dis. 2022 Nov 25;13(11):997. doi: 10.1038/s41419-022-05431-2 (PMC9700717; doi:10.1038/s41419-022-05431-2)
Supplement: Supplementary file 1 — Supplementary information [file 41419_2022_5431_MOESM1_ESM.pdf]

Supplementary Information

Translational readthrough of nonsense mutant TP53 by mRNA incorporation of 5-Fluorouridine

This file includes:

Supplementary Figures S1-S7

Supplementary Tables S1-S2

Figure S1

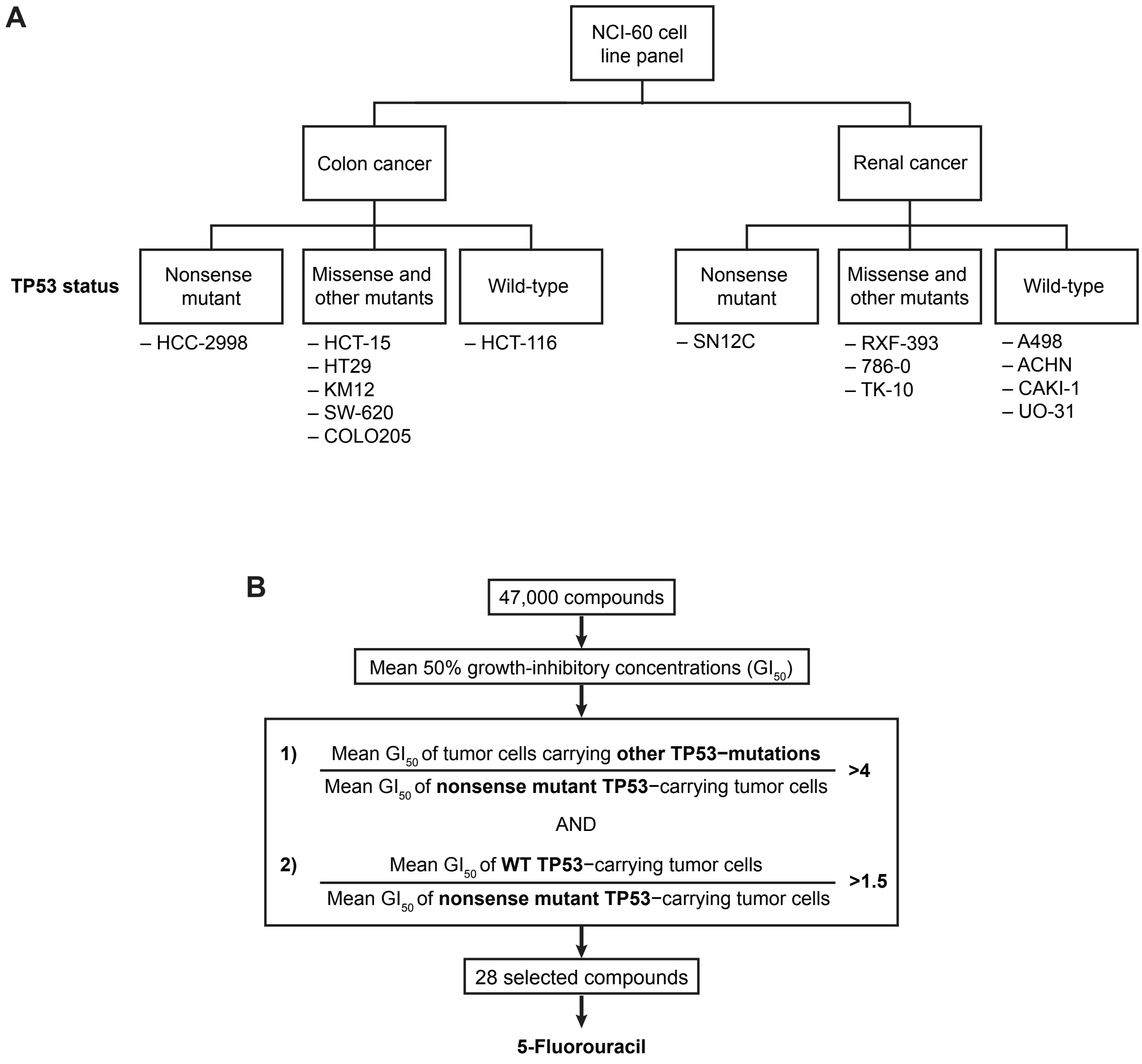

Figure S1. Schematic description of the NCI-60 database data analysis. A, Cell lines included in the analysis divided by cancer type and *TP53* status. **B,** Ratio calculations of the mean 50% growth-inhibitory concentrations (GI_{50}) for all compounds in each cell line group and criteria chosen to select positive hit compounds.

Figure S2

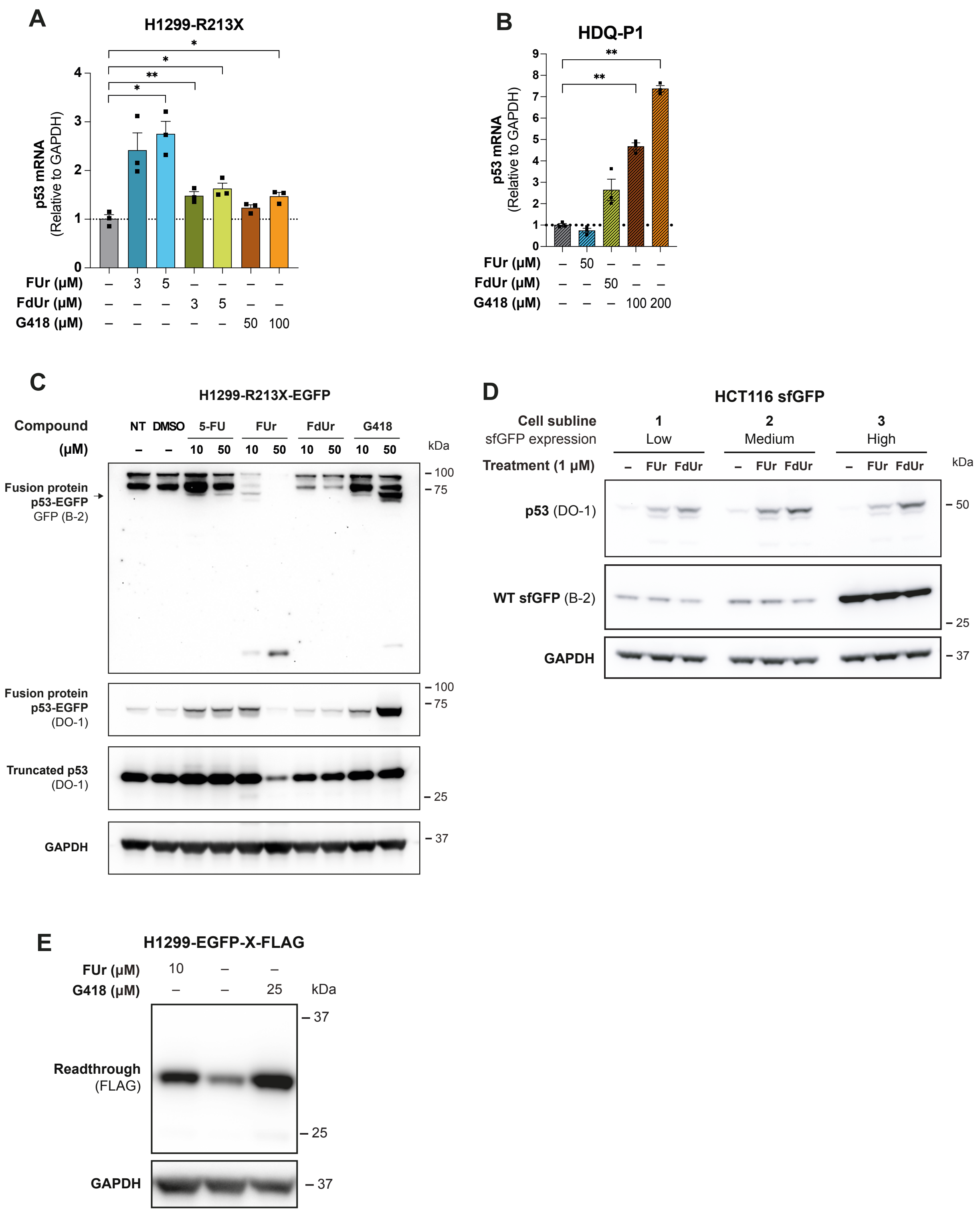

Figure S2. Effect of FUr and FdUr on *TP53* mRNA and WT sfGFP. A, qRT-PCR analysis of *TP53* mRNA levels after treatment with 3 or 5 μM FUr or FdUr or 50 or 100 μM G418 for 72 h in H1299-R213X cells. N=3. **B,** qRT-PCR analysis of *TP53* mRNA levels after treatment with 50 μM FUr or FdUr or 100 or 200 μM G418 for 72 h in HDQ-P1 cells. N=3. **C,** Western blot analysis

of H1299-R213X-EGFP cells treated with different concentrations of 5-FU, FUr, FdUr or G418 for 72h. Fusion protein consisting of full-length p53 and EGFP was detected with GFP antibody B-2 and with p53 antibody DO-1. Truncated p53 was also detected with p53 antibody DO-1. GAPDH was used as loading control and DMSO or untreated cells (-) were used as negative controls. Full membrane was blotted with GFP antibody B-2, then stripped, blocked again and blotted with p53 antibody DO-1. Membrane was then washed and blotted with GAPDH antibody. **D,** Western blot analysis of three HCT116 cell sublines stably transfected with wild-type (WT) superfolder GFP (sfGFP) construct, which express low, medium or high sfGFP levels. WT sfGFP is detected by GFP antibody B-2 and WT p53 is detected with p53 antibody DO-1. Cells were treated with 1 μM FUr or FdUr for 24 h. GAPDH was used as loading control. Full membrane was blotted with GFP antibody, washed and blotted with DO-1 antibody, and washed and blotted with GAPDH antibody. **E,** Western blot analysis of H1299-EGFP-X-FLAG cells treated with 10 μM FUr or 25 μM of G418 for 72 h. Readthrough induction was detected by FLAG antibody. GAPDH was used as loading control and DMSO (-) was used as a negative control. Full membrane was blotted with FLAG antibody, washed and blotted with GAPDH antibody.

In **A** and **B**, each dot represents an independent experiment. Data are represented as mean ± SEM. Values were normalized to GAPDH expression and to the untreated sample (NT). Statistical analyses were performed comparing each treatment to only the NT sample using the repeated measures one-way ANOVA followed by Dunnett's multiple comparisons test (*$p \leq 0.05$, **$p \leq 0.01$). Note that in **A**, expression of p53 was examined simultaneously with other genes presented in Figure 5C and in **B** with genes presented in Figure 5E, with a single GAPDH control. Thus, same GAPDH value was used as control for several genes.

Figure S3

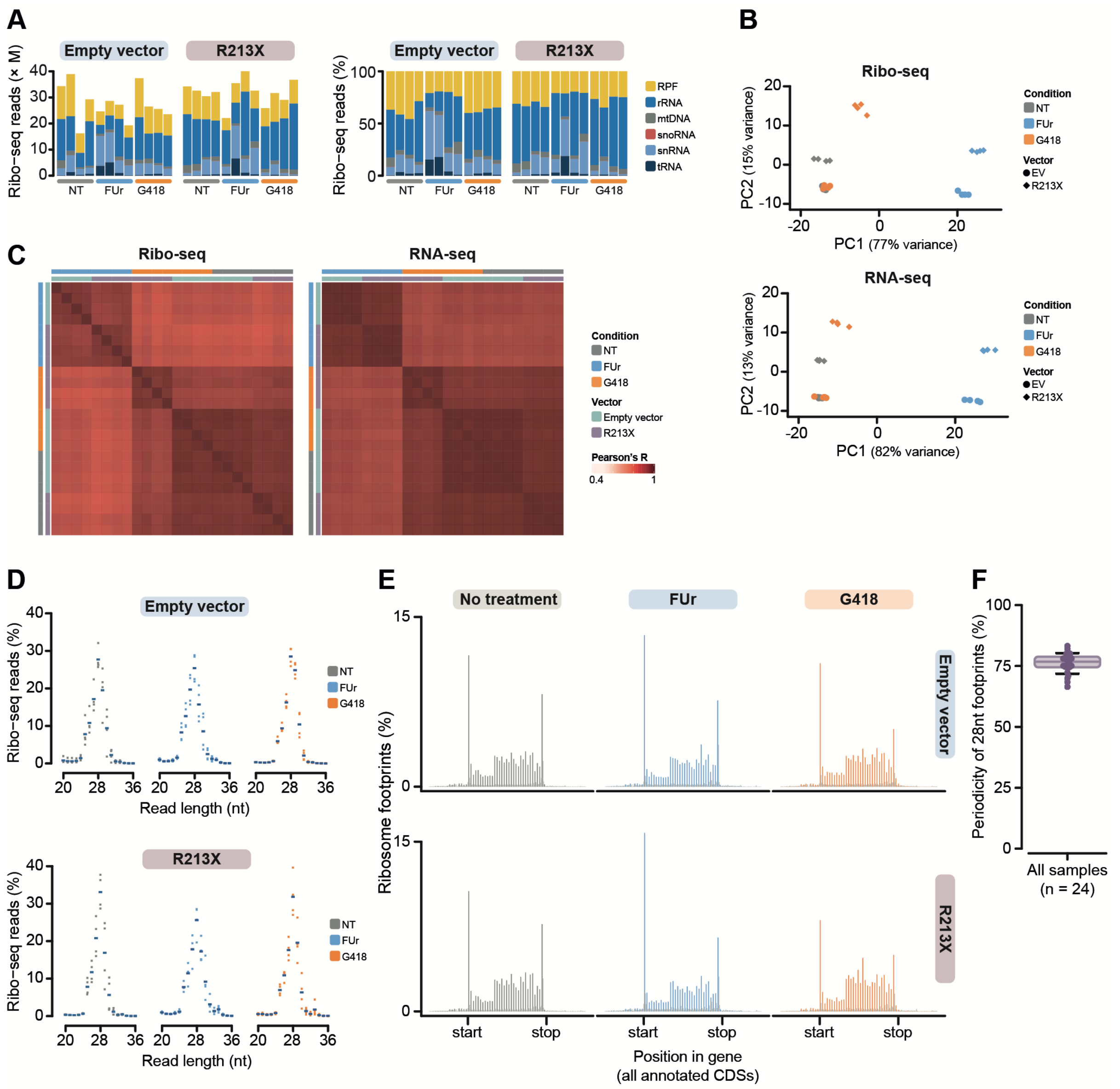

Figure S3. Ribo-seq quality control analysis. A, Stacked bar plots displaying the number (left) and fraction (right) of ribosomal footprint (RPF) reads, as well as reads mapping to common sources of Ribo-seq data 'contaminants' including tRNA, rRNA, mitochondrial DNA, snoRNA, and snRNA. **B,** Principal component analysis based on the top-500 protein-coding genes with the highest variance for Ribo-seq (top) and RNA-seq (bottom) libraries, to illustrate the high technical replicability of treatment and vector types. **C,** Heatmaps showing inter-sample Pearson correlations. Colored annotations demarcate treatment and the vector type (empty vector or vector carrying the *TP53* coding sequence with the R213X mutation) for each sample. **D,** Histogram showing the distribution of read lengths in Ribo-seq experiments. Horizontal blue

bars denote the mean per read length across 4 technical replicates. **E,** Metagene periodicity bar plots of Ribo-seq data. Each colored bar represents bins with in-frame P-sites. Percentages indicate the fraction of all reads in that particular bin. The 4 technical replicates were merged for this visualization. **F,** Dot plot showing the percentage of in-frame reads using read length of 28 nt as reference. Each dot represents a single sample. Data are represented as median ± 95% confidence interval, box limits represent the upper and lower quartiles.

Figure S4

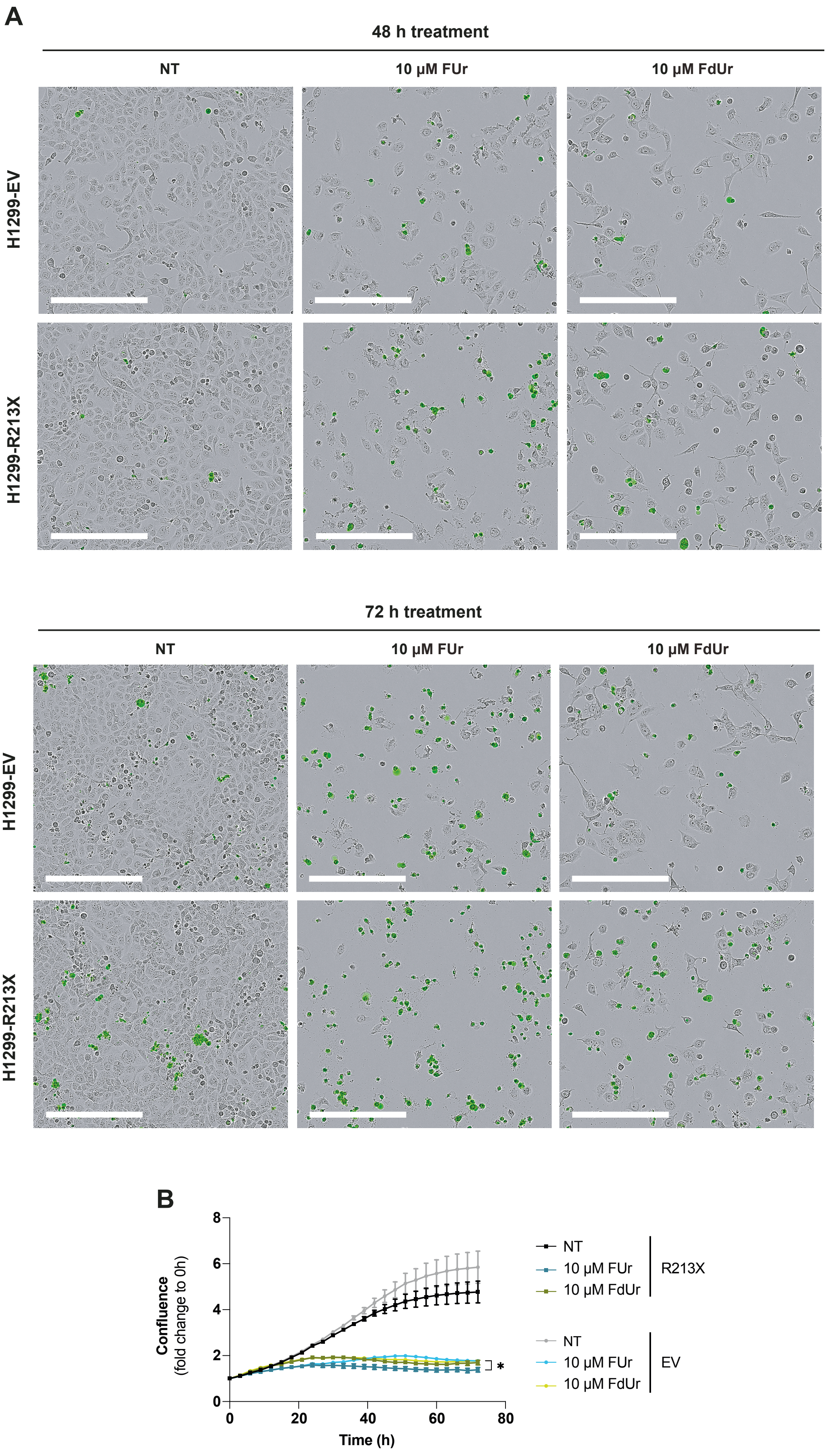

A
48 h treatment
NT
10 µM FUr
10 µM FdUr
H1299-EV
H1299-R213X
72 h treatment
NT
10 µM FUr
10 µM FdUr
H1299-EV
H1299-R213X
B
Confluence
(fold change to 0h)
8
6
4
2
0
0
20
40
60
80
Time (h)
*
NT
10 µM FUr
10 µM FdUr
R213X
NT
10 µM FUr
10 µM FdUr
EV

C

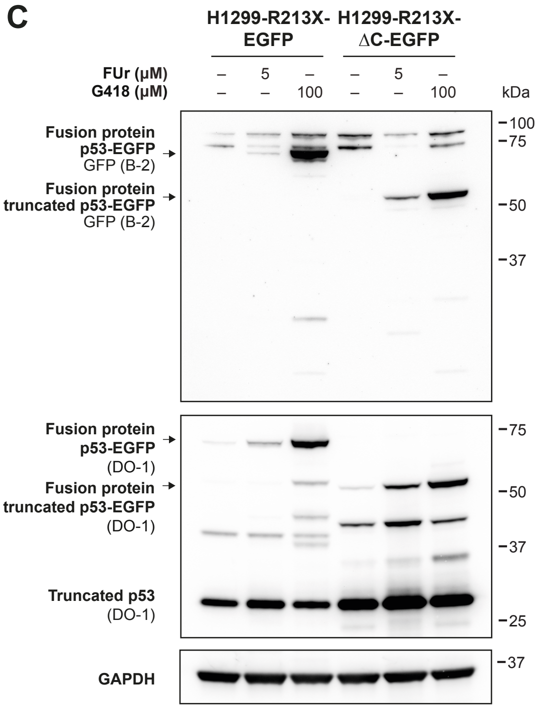
H1299-R213X-EGFP
H1299-R213X-ΔC-EGFP
FUr (μM)
G418 (μM)
– 5 – – 5 –
– – 100 – – 100
kDa
Fusion protein p53-EGFP GFP (B-2)
Fusion protein truncated p53-EGFP GFP (B-2)
100
75
50
37
Fusion protein p53-EGFP (DO-1)
Fusion protein truncated p53-EGFP (DO-1)
Truncated p53 (DO-1)
75
50
37
25
GAPDH
37

D

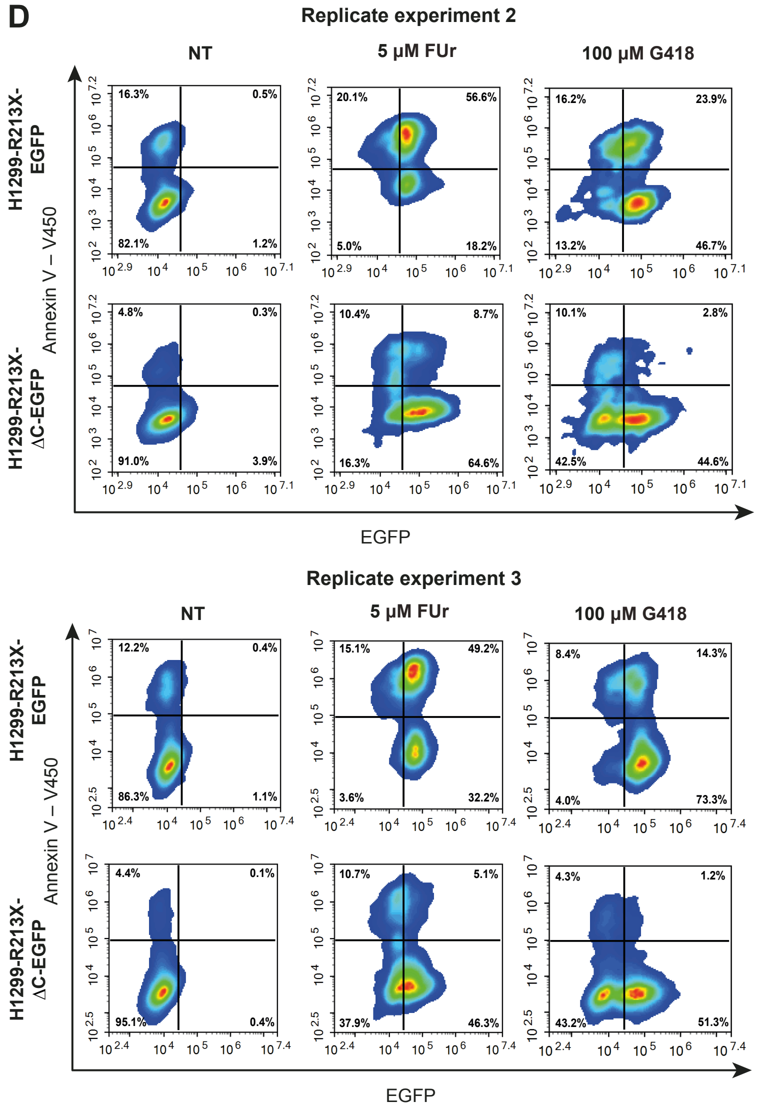
Replicate experiment 2
NT
5 μM FUr
100 μM G418
H1299-R213X-EGFP
H1299-R213X-ΔC-EGFP
Annexin V – V450
16.3%
0.5%
82.1%
1.2%
20.1%
56.6%
5.0%
18.2%
16.2%
23.9%
13.2%
46.7%
4.8%
0.3%
91.0%
3.9%
10.4%
8.7%
16.3%
64.6%
10.1%
2.8%
42.5%
44.6%
EGFP
Replicate experiment 3
NT
5 μM FUr
100 μM G418
H1299-R213X-EGFP
H1299-R213X-ΔC-EGFP
Annexin V – V450
12.2%
0.4%
86.3%
1.1%
15.1%
49.2%
3.6%
32.2%
8.4%
14.3%
4.0%
73.3%
4.4%
0.1%
95.1%
0.4%
10.7%
5.1%
37.9%
46.3%
4.3%
1.2%
43.2%
51.3%
EGFP

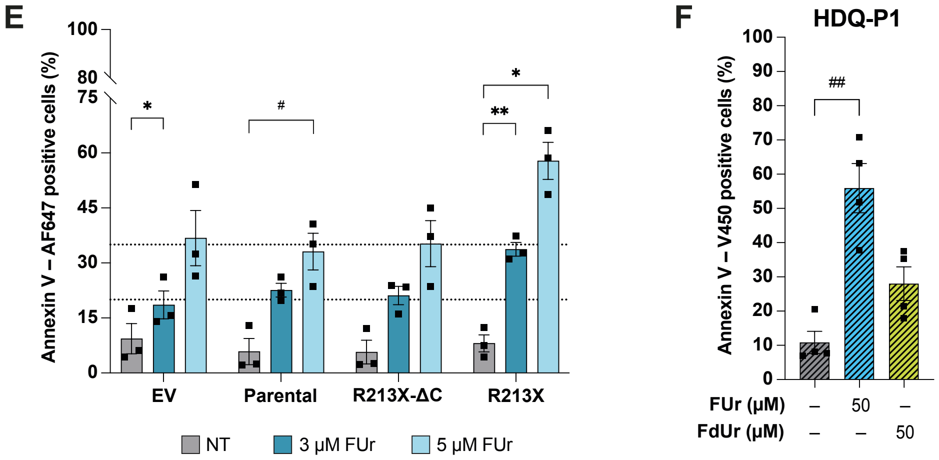

Figure S4. FUr induces R213X p53-dependent cell death. A, Representative images of data presented in Figure 4B, showing caspase 3/7 cleavage activity in H1299-R213X and H1299-EV cells after treatment with 10 μM FUr or FdUr up to 72 h. Images correspond to 48 h (top panel) and 72 h (bottom panel) after treatment. Images were obtained with Incucyte® S3 system at 10X magnification. Green cells correspond to cells with caspase 3/7 cleavage activity. Scale bar = 400 μm. **B,** Cell confluence curves of H1299-R213X and H1299-EV cells after treatment with 10 μM FUr or FdUr up to 72 h. Data are presented as fold change to 0 h time point. N=3-4, data are represented as mean ± SEM. Differences between the two cell lines within each treatment group at the 72 h treatment time point were analyzed using independent *t*-test, p = 0.0182 (R213X 10 μM FUr vs. EV 10 μM FUr). **C,** Western blot analysis of H1299-R213X-EGFP and H1299-R213X-ΔC-EGFP cells treated with 5 μM FUr or 100 μM G418 for 72h. The full-length p53-EGFP fusion protein in H1299-R213X-EGFP cells and the truncated p53-EGFP fusion protein in H1299-R213X-ΔC-EGFP cells were detected with GFP antibody B-2 and with p53 antibody DO-1. Truncated p53 was also detected with p53 antibody DO-1. GAPDH was used as loading control and untreated cells (-) were used as negative controls. Full membrane was blotted with GFP antibody B-2, then stripped, blocked again and blotted with p53 antibody DO-1. Membrane was then washed and blotted with GAPDH antibody. **D,** Replicates 2 and 3 from Annexin V staining and EGFP expression assessed by flow cytometry in H1299-R213X-EGFP and H1299-R213X-ΔC-EGFP cells after 5 μM FUr or 100 μM G418 treatment for 72 h (Figure 4C). **E,** Annexin V staining analysis by flow cytometry of H1299-EV (EV), H1299 parental

(Parental), H1299-R213X-ΔC-FLAG (R213X-ΔC) and H1299-R213X (R213X) cells treated with 3 or 5 µM FUr for 72 h. N=3, data are represented as mean ± SEM. Differences between 3 or 5 µM vs. non-treated (NT) sample within each cell line were analyzed with repeated measures one-way ANOVA followed by Dunnett's multiple comparisons test ($^{*}p \leq 0.05$, $^{**}p \leq 0.01$) or Friedman test followed by Dunn's multiple comparisons test ($^{\#}p \leq 0.05$) in H1299 parental (Parental) cells as data did not fit normal distribution. Dashed lines at Y=20 and Y=35 are shown as reference. **F,** Annexin V staining analysis by flow cytometry of HDQ-P1 cells treated with 50 µM FUr or FdUr for 72 h. N=4, data are represented as mean ± SEM. Since a p53 null subline of HDQ-P1 is not available, fluorescence obtained with the same treatment in unstained cells was subtracted. Differences between 50 µM FUr or FdUr vs. non-treated (-) sample were analyzed with Friedman test followed by Dunn's multiple comparisons test ($^{\#\#}p \leq 0.01$) as data did not fit normal distribution.

Figure S5

A

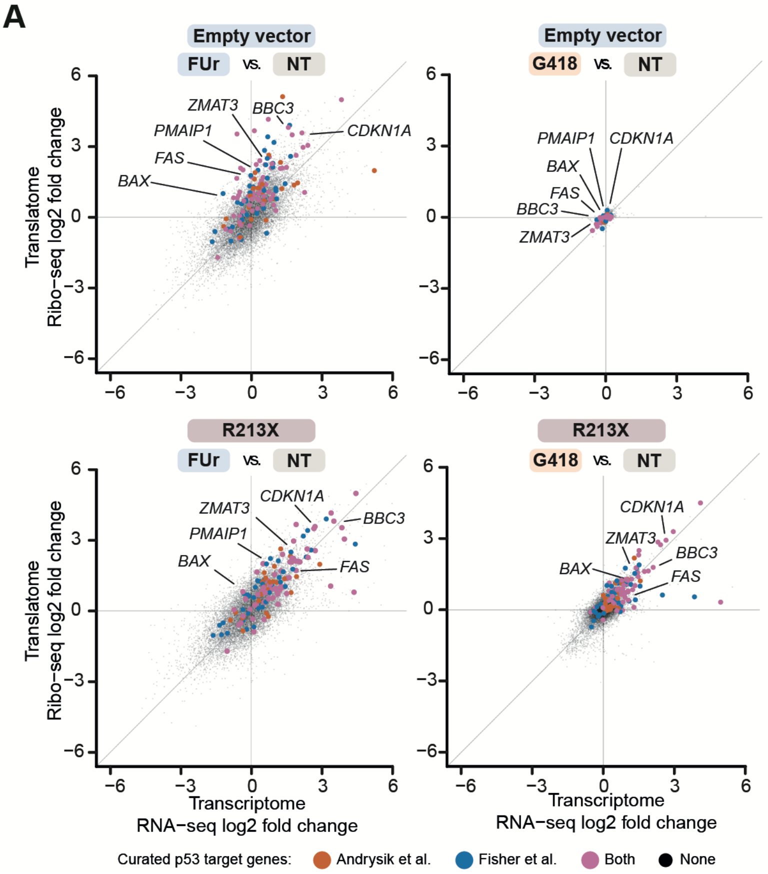

Empty vector
FUr vs. NT
Empty vector
G418 vs. NT
R213X
FUr vs. NT
R213X
G418 vs. NT
ZMAT3
BBC3
PMAIP1
CDKN1A
FAS
BAX
Translatome
Ribo−seq log2 fold change
Transcriptome
RNA−seq log2 fold change
Curated p53 target genes:
Andrysik et al.
Fisher et al.
Both
None

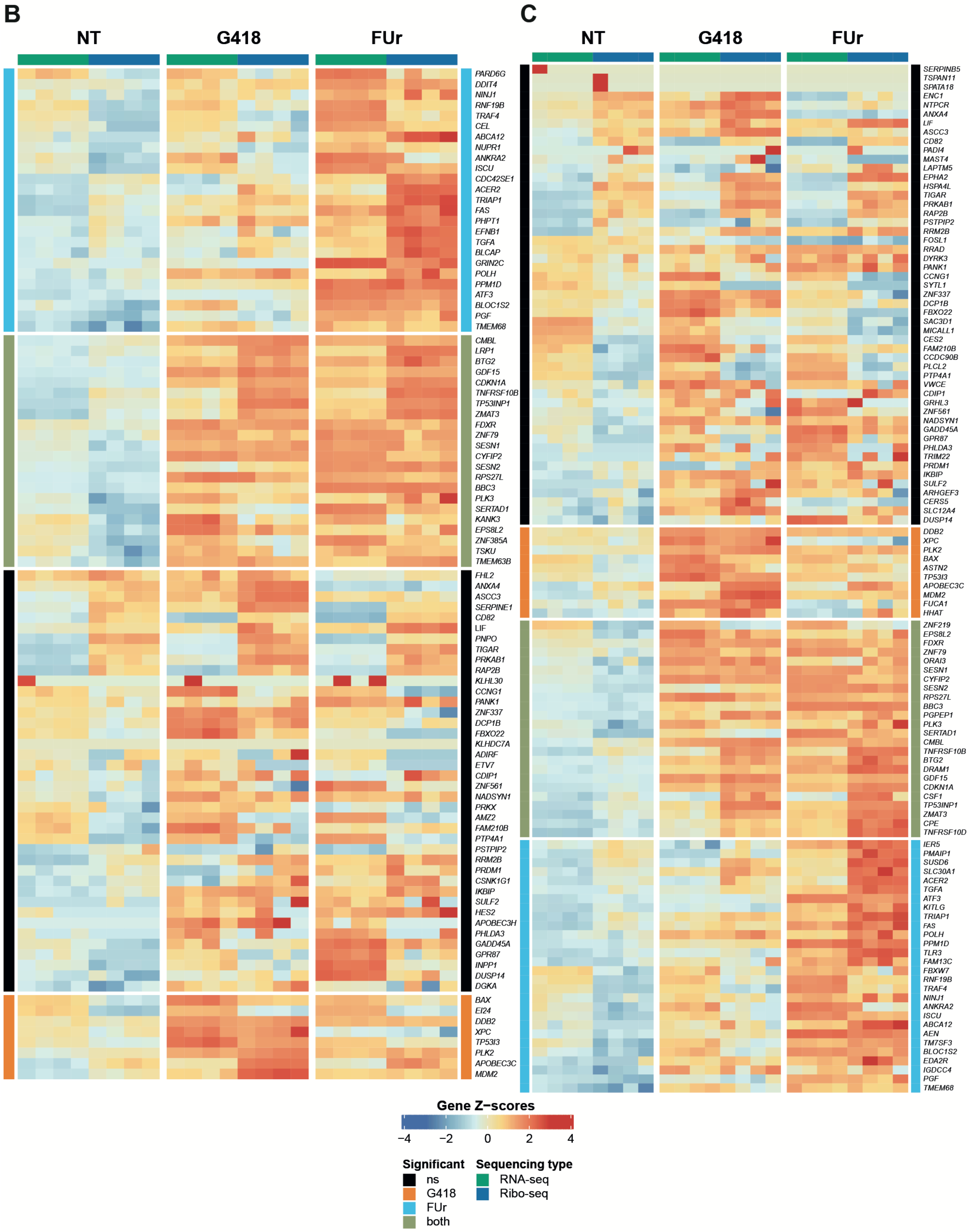
B
NT
G418
FUr
PARD6G
DDIT4
NINJ1
RNF19B
TRAF4
CEL
ABCA12
NUPR1
ANKRA2
ISCU
CDC42SE1
ACER2
TRIAP1
FAS
PHPT1
EFNB1
TGFA
BLCAP
GRIN2C
POLH
PPM1D
ATF3
BLOC1S2
PGF
TMEM68
CMBL
LRP1
BTG2
GDF15
CDKN1A
TNFRSF10B
TP53INP1
ZMAT3
FDXR
ZNF79
SESN1
CYFIP2
SESN2
RPS27L
BBC3
PLK3
SERTAD1
KANK3
EPS8L2
ZNF385A
TSKU
TMEM63B
FHL2
ANXA4
ASCC3
SERPINE1
CD82
LIF
PNPO
TIGAR
PRKAB1
RAP2B
KLHL30
CCNG1
PANK1
ZNF337
DCP1B
FBXO22
KLHDC7A
ADIRF
ETV7
CDIP1
ZNF561
NADSYN1
PRKX
AMZ2
FAM210B
PTP4A1
PSTPIP2
RRM2B
PRDM1
CSNK1G1
IKBIP
SULF2
HES2
APOBEC3H
PHLDA3
GADD45A
GPR87
INPP1
DUSP14
DGKA
BAX
EI24
DDB2
XPC
TP53I3
PLK2
APOBEC3C
MDM2
C
NT
G418
FUr
SERPINB5
TSPAN11
SPATA18
ENC1
NTPCR
ANXA4
LIF
ASCC3
CD82
PADI4
MAST4
LAPTM5
EPHA2
HSPA4L
TIGAR
PRKAB1
RAP2B
PSTPIP2
RRM2B
FOSL1
RRAD
DYRK3
PANK1
CCNG1
SYTL1
ZNF337
DCP1B
FBXO22
SAC3D1
MICALL1
CES2
FAM210B
CCDC90B
PLCL2
PTP4A1
VWCE
CDIP1
GRHL3
ZNF561
NADSYN1
GADD45A
GPR87
PHLDA3
TRIM22
PRDM1
IKBIP
SULF2
ARHGEF3
CERS5
SLC12A4
DUSP14
DDB2
XPC
PLK2
BAX
ASTN2
TP53I3
APOBEC3C
MDM2
FUCA1
HHAT
ZNF219
EPS8L2
FDXR
ZNF79
ORAI3
SESN1
CYFIP2
SESN2
RPS27L
BBC3
PGPEP1
PLK3
SERTAD1
CMBL
TNFRSF10B
BTG2
DRAM1
GDF15
CDKN1A
CSF1
TP53INP1
ZMAT3
CPE
TNFRSF10D
IER5
PMAIP1
SUSD6
SLC30A1
ACER2
TGFA
ATF3
KITLG
TRIAP1
FAS
POLH
PPM1D
TLR3
FAM13C
FBXW7
RNF19B
TRAF4
NINJ1
ANKRA2
ISCU
ABCA12
AEN
TM7SF3
BLOC1S2
EDA2R
IGDCC4
PGF
TMEM68
Gene Z−scores
−4
−2
0
2
4
Significant
ns
G418
FUr
both
Sequencing type
RNA-seq
Ribo-seq

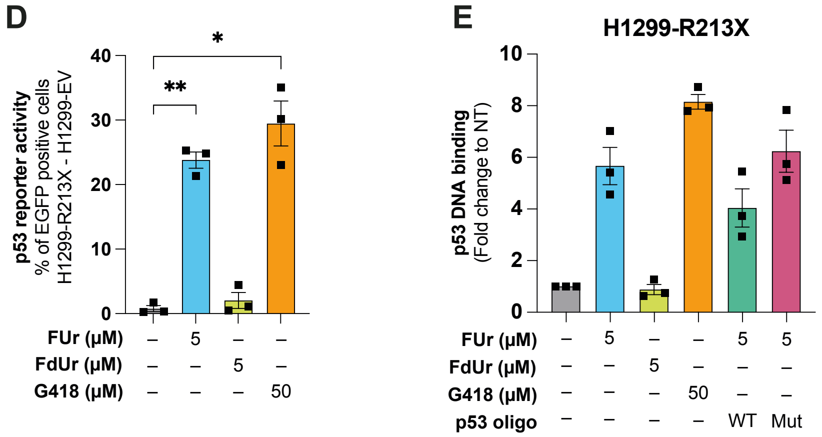

Figure S5. FUr-induced full-length p53 is transcriptionally active. A, RNA-seq to Ribo-seq log2 fold-change/fold-change (FC/FC) plots showing differentially transcribed (x axis) and translated (y axis) genes in H1299-EV (top panels) and H1299-R213X (R213X) (bottom panels) cells for FUr (left panels) and G418 (right panels) treatments. Each dot on each graph represents a protein-coding gene. Curated *TP53* target genes from Andrysik *et al.*[1] are coloured red and curated *TP53* target genes from Fischer[2] are in blue. Genes found in both lists are coloured purple. Selected *TP53* target genes validated by qPCR in (Figure 5C-D) are labelled by gene symbol, *CDKN1A* (p21), *ZMAT3* (Zmat3), *BBC3* (Puma), *PMAIP1* (Noxa), *FAS* (Fas) and *BAX* (Bax). **B** and **C,** Heatmap showing gene expression of *TP53* target gene lists as obtained from Andrysik *et al.*[1] **(B)** and from Fischer[2] **(C)** organized per treatment condition in H1299-R213X cells, as measured by RNA-seq (green, left) and Ribo-seq (blue, right). Statistical analyses for each treatment were performed using DESeq2 (ref. 3), i.e., a Wald test with Benjamin-Hochberg multiple test correction (adjusted *p*-value ≤ 0.05, log2 fold change ≥ 1) to test for significant genes in Ribo-seq data, colours indicate differentially expressed genes. Normalised counts were transformed to Z-scores for visualisation. **D,** Flow cytometry analysis of p53-EGFP reporter activity after 5 µM FUr or FdUr or 50 µM G418 treatment for 72 h. Activity of p53 as transcription factor was assessed by percentage of EGFP positive cells in H1299-R213X from which EGFP positive cells in H1299-EV cells were subtracted, N=3. Differences between each treatment vs. non-treated (-) sample were analyzed with repeated measures one-way ANOVA followed by Dunnett's multiple comparisons test (*$p \leq 0.05$, **$p \leq 0.01$). **E,** p53 DNA binding activity

measured by DNA binding ELISA (TransAM p53) in H1299-R213X cells treated with 5 μM FUr or FdUr or 50 μM G418 treatment for 72 h. Competition with WT or mutant (Mut) p53 oligos were used to assess DNA binding specificity. Fold change of each sample to non-treated (-) control was calculated for each independent experiment, N=3.

Figure S6

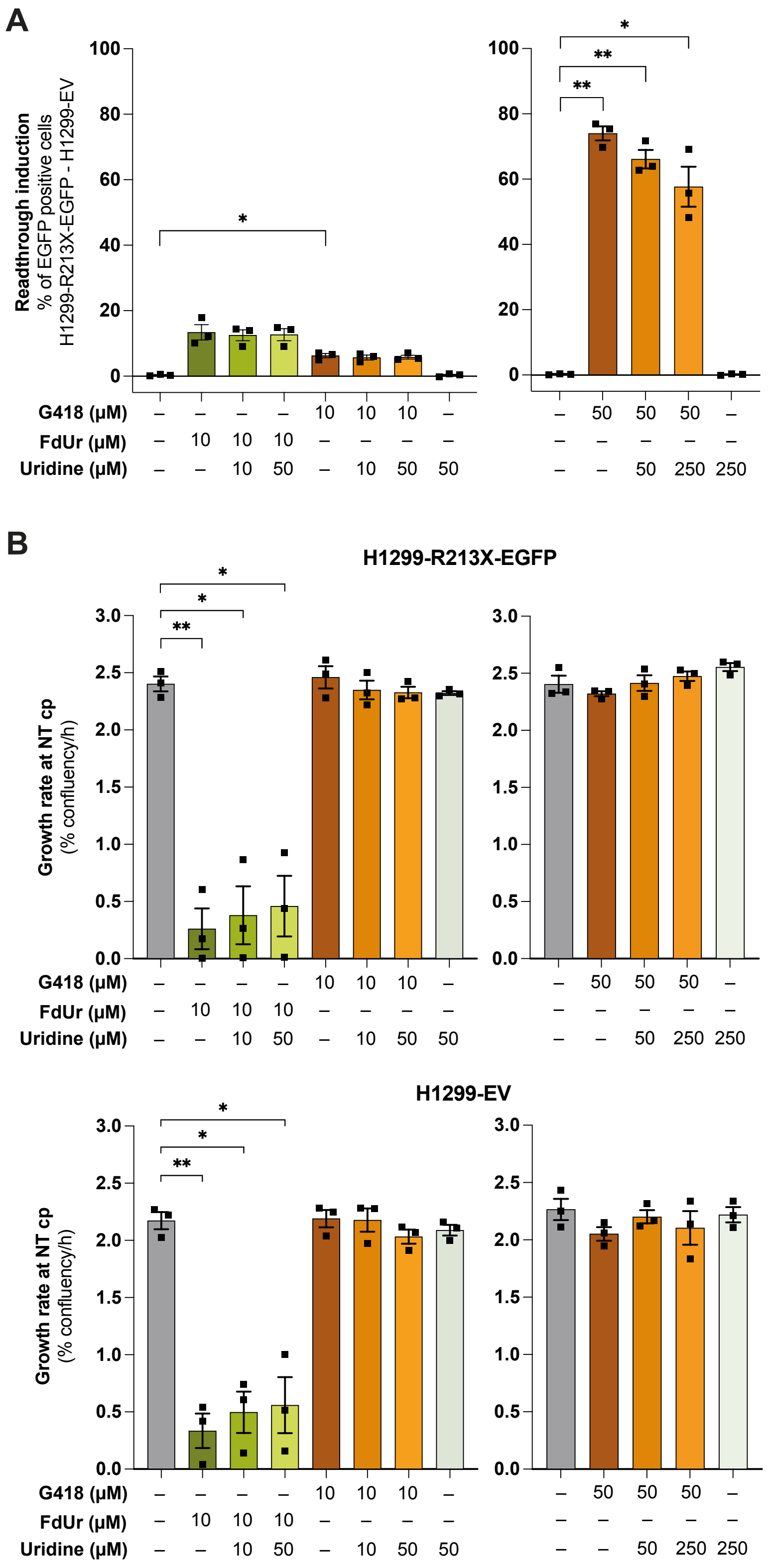

Figure S6. Uridine competes with FUr and attenuates readthrough of R213X nonsense mutant *TP53*. A, Flow cytometry analysis of readthrough induction after 10 μM FdUr or G418 treatment alone or in combination with 10 or 50 μM uridine or 50 μM uridine alone (left panel) or

after 50 µM G418 treatment alone or in combination with 50 or 250 µM uridine or 250 µM uridine alone (right panel) for 72 h. Readthrough induction was assessed by percentage of EGFP positive cells in H1299-R213X-EGFP from which EGFP positive cells in H1299-EV cells were subtracted, N=3. Note that results in left panel were obtained in two of the experiments presented in Figure 6C with FUr treatment, so values for two non-treated samples (NT) are the same in both figures. **B,** Cell growth rate of H1299-R213X-EGFP (top panel) or H1299-EV (bottom panel) cells treated with 10 µM FdUr or G418 treatment alone or in combination with 10 or 50 µM uridine or 50 µM uridine alone (left panel) or 50 µM G418 treatment alone or in combination with 50 or 250 µM uridine or 250 µM uridine alone (right panel) monitored up to 72 h with Incucyte® S3 system. Data are presented as cell growth rate in percentage of confluency per hour when growth rate of the non-treated cell population (-) reaches its maximum or critical point (cp), N=3. Note that results in left panels were obtained in two of the experiments presented in Figure 6E with FUr treatment, and so values for two non-treated samples (NT) are the same in both figures.

Data are represented as mean ± SEM. Differences between all conditions were analyzed using repeated measures one-way ANOVA followed by Tukey's multiple comparisons test (*$p \leq 0.05$, **$p \leq 0.01$) or Friedman test followed by Dunn's multiple comparisons test for data not fitting normal distribution. The differences shown are between each treatment vs. the non-treated sample (NT) and between each treatment alone (FdUr or G418) vs. its each combination treatment with uridine.

Figure S7

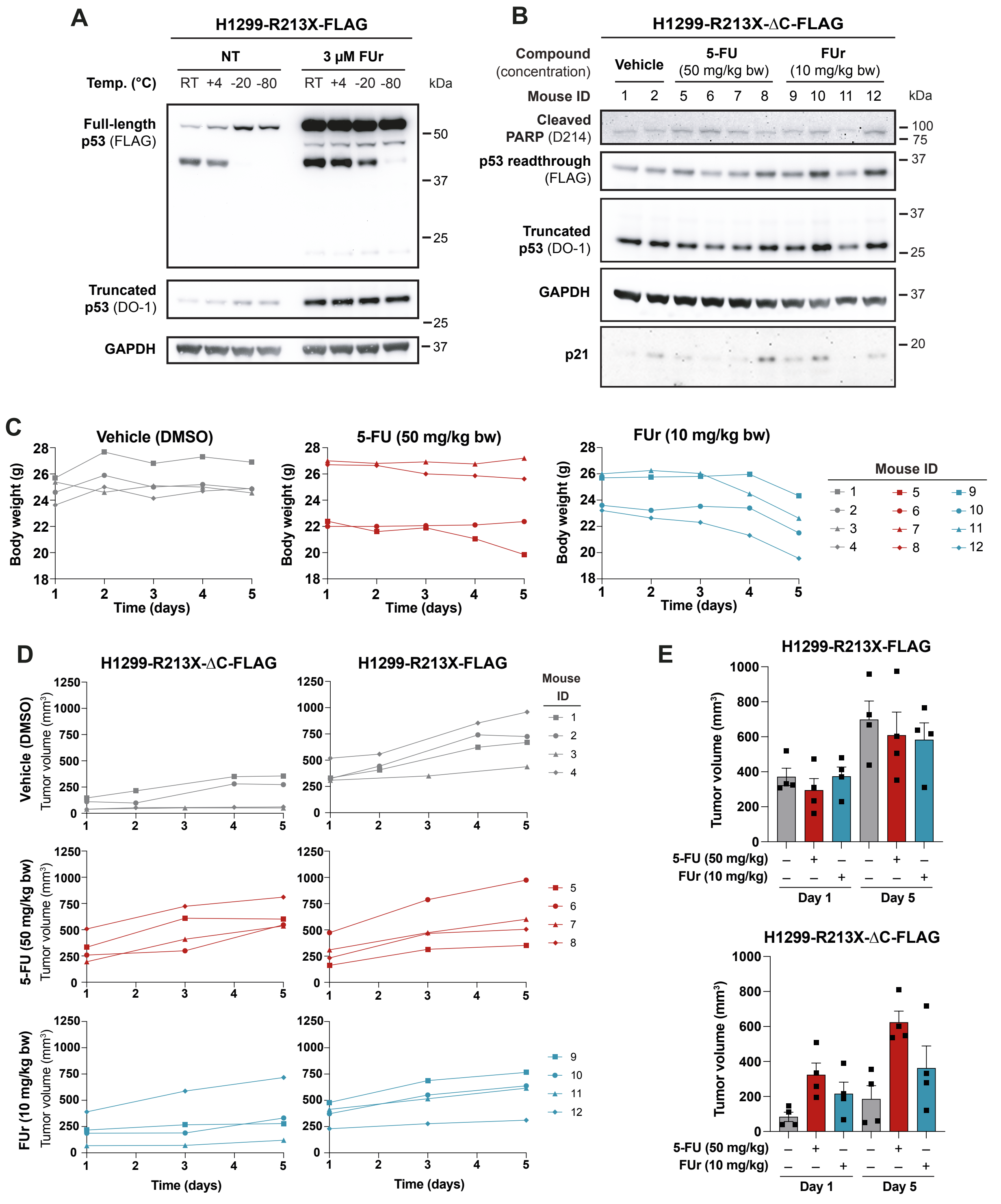

Figure S7. 5-FU and FUr upregulate full-length p53 *in vivo*. A, Western blot analysis of H1299-R213X-FLAG cells non-treated (NT) or treated with 3 µM FUr for which lysates were kept at room temperature (RT), +4°C, -20°C or -80°C for 3 days to assess p53 protein degradation. Full-length p53 was detected with FLAG antibody after a long exposure (saturated image) to

visualize low abundance degradation products. After FLAG detection, membrane was washed and blotted with p53 antibody DO-1 to detect truncated p53. GAPDH was used as loading control. The band above 37 kD was not detected after incubation at -80°C but appeared with increasing intensity when protein lysates were incubated at higher temperatures, supporting the assumption that it is a p53 degradation product. **B,** Western blot analysis of H1299-R213X-ΔC-FLAG xenograft tumors from mice treated with 50 mg/kg of body weight (mg/kg bw) of 5-FU or 10 mg/kg bw of FUr or with vehicle (DMSO), as a negative control, for 5 days. Readthrough induction was detected with FLAG antibody and truncated p53 stabilization with p53 antibody DO-1. Cleaved PARP was detected with Cleaved PARP D214 antibody and p21 was detected with p21 F-5 antibody. GAPDH was used as loading control. Membrane was cut at 75 and 25 kDa. Upper part was blotted with Cleaved PARP antibody. Middle part was blotted with FLAG antibody, then washed and blotted with DO-1 antibody, washed and blotted with GAPDH antibody. Bottom part was blotted with p21 antibody. **C,** Mice body weight over indicated treatments. Each line corresponds to one mouse. **D,** Tumor volumes (mm^3) of H1299-R213X-ΔC-FLAG (left panels) and H1299-R213X-FLAG (right panels) xenografts upon treatment with DMSO (top panels), 5-FU (middle panels) or FUr (bottom panels). Each line represents one mouse. **E,** Bar chart of data shown in panel D with tumor volumes (mm^3) of H1299-R213X-FLAG (top panel) and H1299-R213X-ΔC-FLAG (lower panel) xenografts at first day of treatment (day 1) and last day of treatment (day 5). Each dot represents one mouse. Data is expressed as the mean from 4 mice in each group ± SEM.

Table S1. Cellular uptake of 5-FU, FUr, FdUr and G418. Free intracellular (upper table) and extracellular (lower table) levels of G418, 5-FU, FUr or FdUr after treatment with 50 µM of each compound for 1, 2 or 3 days. Data are expressed as pM per cell for intracellular and µM for extracellular levels, respectively. Values in bold indicate levels of the specified compound upon treatment with the same compound.

		Free intracellular levels (pM/cell)			
**Compound**	**Treatment (days)**	**c-G418**	**c-5-FU**	**c-FUr**	**c-FdUr**
G418	1	**0.00**	0.00	0.00	0.00
G418	2	**0.00**	0.00	0.00	0.00
G418	3	**0.03**	0.00	0.00	0.00
5-FU	1	0.00	**5.07**	1.74	0.03
5-FU	2	0.00	**7.73**	4.43	0.03
5-FU	3	0.00	**5.44**	3.93	0.03
FUr	1	0.00	6.48	**26.83**	0.07
FUr	2	0.00	5.00	**9.65**	0.04
FUr	3	0.00	3.23	**5.09**	0.03
FdUR	1	0.00	2.41	0.65	**0.94**
FdUR	2	0.00	2.99	0.92	**0.26**
FdUR	3	0.00	4.04	1.39	**0.18**

		Extracellular levels (µM)			
**Compound**	**Treatment (days)**	**m-G418**	**m-5-FU**	**m-FUr**	**m-FdUr**
G418	0	**50.000**	0.000	0.000	0.000
G418	1	**43.138**	0.000	0.000	0.000
G418	2	**29.856**	0.000	0.000	0.000
G418	3	**21.112**	0.000	0.000	0.000
5-FU	0	0.000	**50.000**	0.000	0.000
5-FU	1	0.000	**48.352**	0.018	0.026
5-FU	2	0.000	**47.304**	0.043	0.030
5-FU	3	0.000	**33.880**	0.045	0.029
FUr	0	0.000	0.030	**50.000**	0.004
FUr	1	0.000	6.317	**28.986**	0.051
FUr	2	0.000	13.506	**27.380**	0.080
FUr	3	0.000	24.993	**28.180**	0.117
FdUR	0	0.000	0.008	0.104	**50.000**
FdUR	1	0.000	2.979	0.021	**40.028**
FdUR	2	0.000	7.481	0.029	**38.564**
FdUR	3	0.000	12.419	0.046	**43.837**

Table S2. Plasmid constructs used for stable transfection of H1299 cells. H1299 parental cells were stably transfected with the indicated constructs as described previously[4]. All constructs were made using a pCMV-puro-bam backbone that contains a CMV promoter and the puromycin resistance gene.

**Name of cell line**	***TP53* mutation**	***TP53* coding sequence**	**Tag or reporter gene fused in frame with *TP53***	**Protein product after readthrough induction**	**Experiments**
H1299-EV	–	–	–	–	Cell culture
H1299-R213X	CGA > TGA (p.R213X)	Full *TP53* coding sequence carrying R213X	–	Full-length p53	Cell culture
H1299-R213X-ΔC-EGFP	CGA > TGA (p.R213X)	*TP53* coding sequence up to R213X	EGFP	Truncated p53 + EGFP	Cell culture
H1299-R213X-EGFP	CGA > TGA (p.R213X)	Full *TP53* coding sequence carrying R213X	EGFP	Full-length p53 + EGFP	Cell culture
H1299-R213X-ΔC-FLAG	CGA > TGA (p.R213X)	*TP53* coding sequence up to R213X	FLAG	Truncated p53 + FLAG	Cell culture and Xenografts *in vivo*
H1299-R213X-FLAG	CGA > TGA (p.R213X)	Full *TP53* coding sequence carrying R213X	FLAG	Full-length p53 + FLAG	Cell culture and Xenografts *in vivo*
H1299-EGFP-X-FLAG	–	–	–	EGFP + FLAG	Cell culture

References

1. Andrysik Z, Galbraith MD, Guarnieri AL, Zaccara S, Sullivan KD, Pandey A, et al. Identification of a core TP53 transcriptional program with highly distributed tumor suppressive activity. Genome Res. 2017;27(10):1645-57.

2. Fischer M. Census and evaluation of p53 target genes. Oncogene. 2017;36(28):3943-56.

3. Love MI, Huber W, Anders S. Moderated estimation of fold change and dispersion for RNA-seq data with DESeq2. Genome Biol. 2014;15(12):550.

4. Zhang M, Heldin A, Palomar-Siles M, Öhlin S, Bykov VJN, Wiman KG. Synergistic Rescue of Nonsense Mutant Tumor Suppressor p53 by Combination Treatment with Aminoglycosides and Mdm2 Inhibitors. Front Oncol. 2018;7:323.
