# Supplementary material for: Translational readthrough of nonsense mutant TP53 by mRNA incorporation of 5-Fluorouridine
Source: Cell Death Dis. 2022 Nov 25;13(11):997. doi: 10.1038/s41419-022-05431-2 (PMC9700717; doi:10.1038/s41419-022-05431-2)
Supplement: Supplementary file 2 — Supplemental material - Original Western blots [file 41419_2022_5431_MOESM2_ESM.pdf]

Original Western blots

Figure 1C

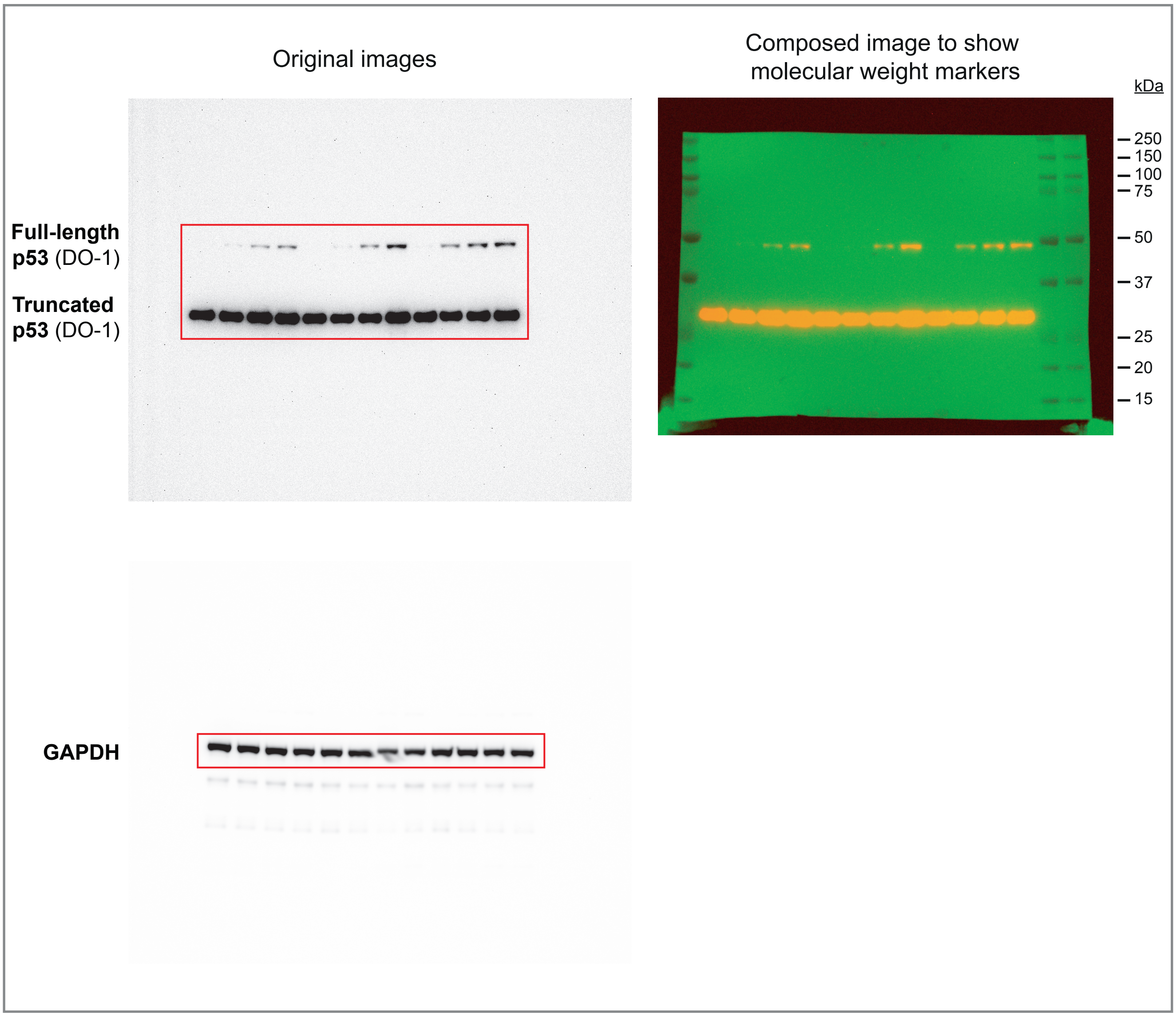

Membrane was first blotted with p53 (DO-1) antibody, washed and blotted with GAPDH, thus some remaining signal from the p53 (DO-1) antibody is observed when developing the GAPDH antibody.

Original Western blots

Figure 2A

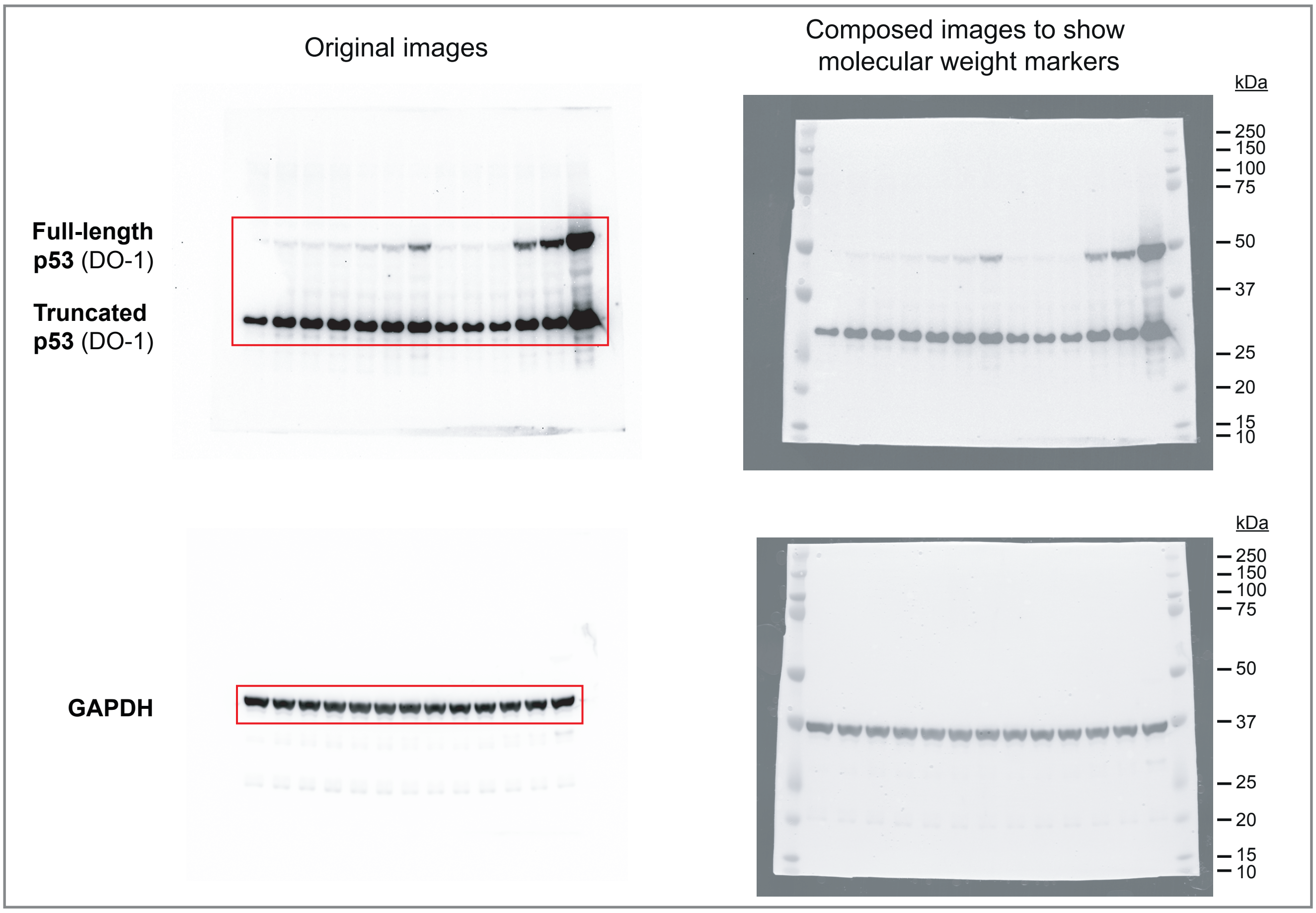

Figure 2B

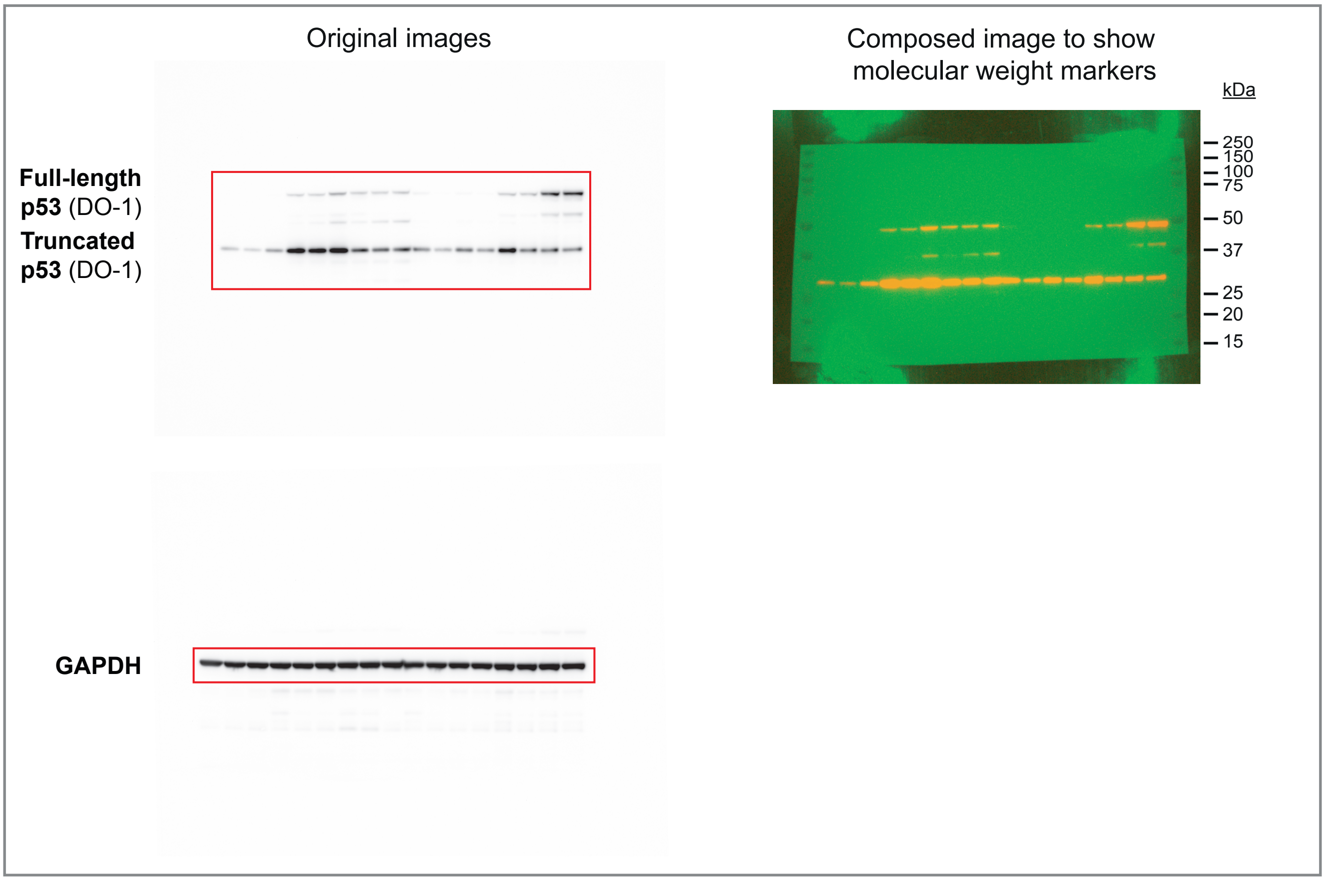

Membranes were first blotted with p53 (DO-1) antibody, washed and blotted with GAPDH, thus some remaining signal from the p53 (DO-1) antibody is observed when developing the GAPDH antibody.

Original Western blots

Figure 2F

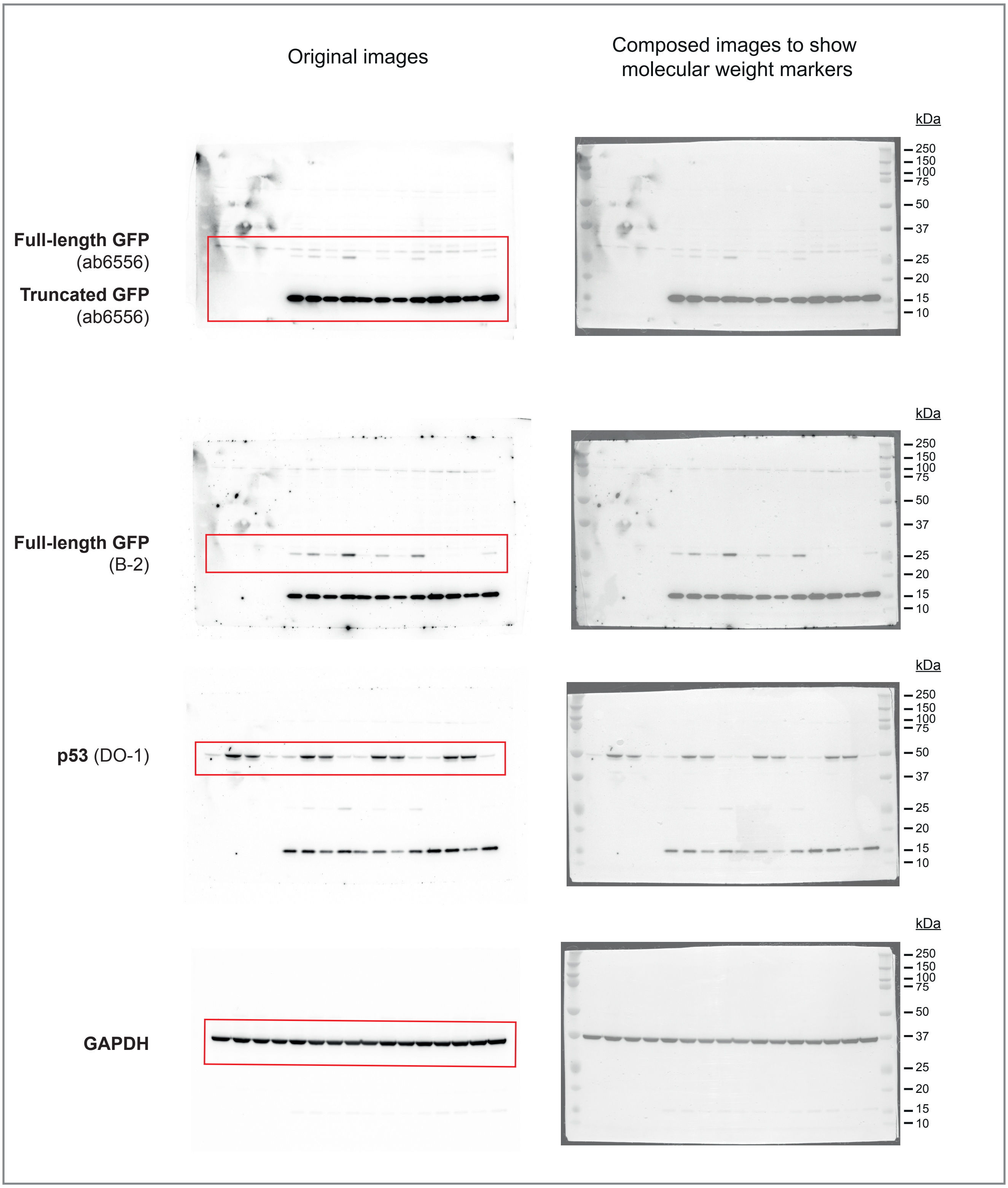

Membrane was first blotted with GFP (ab6556) antibody. Due to strong GFP truncated bands, membrane was washed and blotted with GFP (B-2) antibody, some remaining bands from GFP (ab6556) antibody can be observed. Membrane was washed and blotted with p53 (DO-1) antibody, remaining bands from the two previously used antibodies can be seen. Membrane was lastly blotted with GAPDH antibody.

Original Western blots

Figure 6D

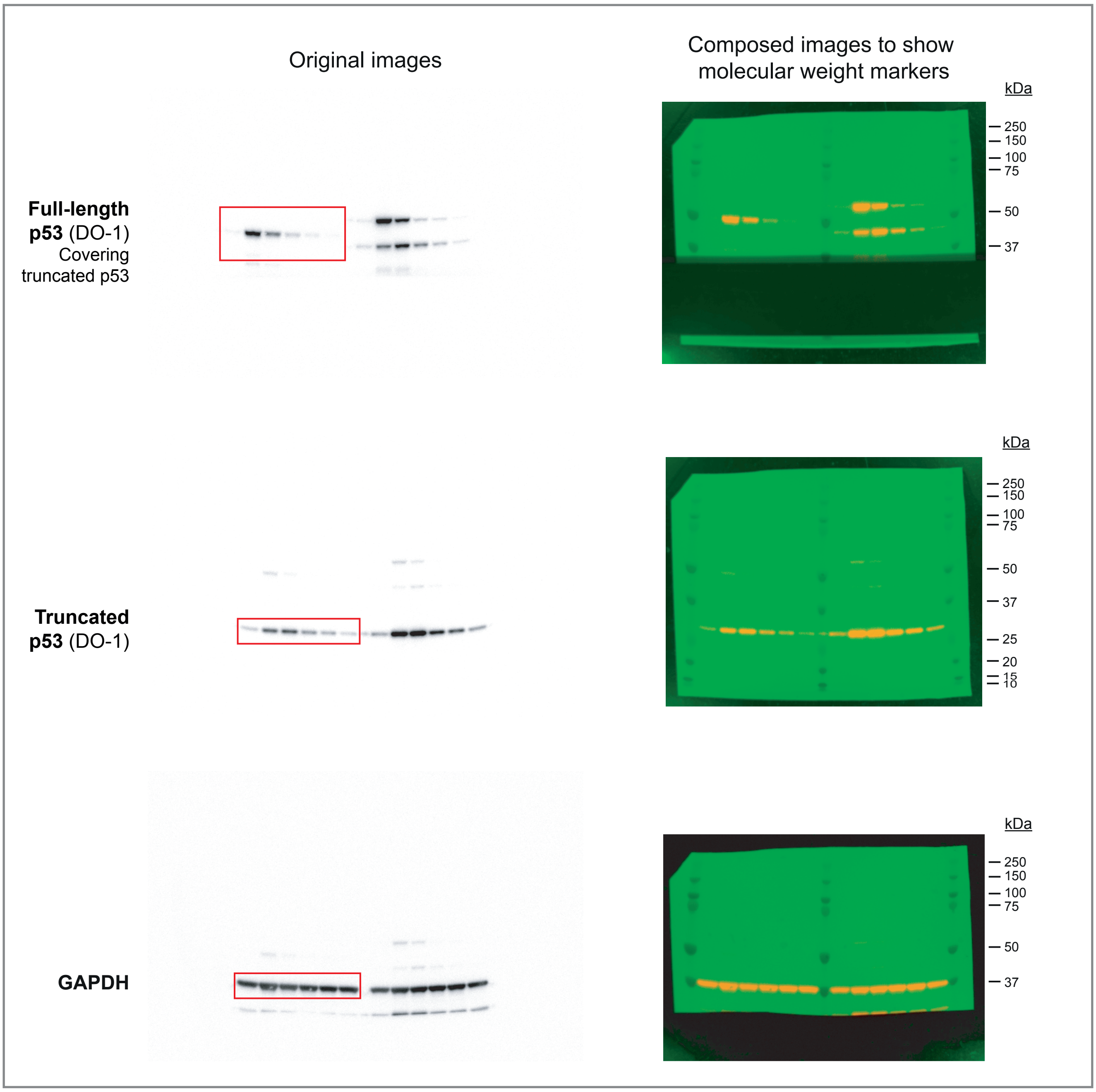

Membrane was first blotted with p53 (DO-1) antibody. First, truncated p53 was visualized. After covering the truncated p53 signal, membrane was exposed again to visualize full-length p53 bands. Finally, membrane was washed and blotted with GAPDH, thus some remaining signal from the p53 (DO-1) antibody is observed when developing the GAPDH antibody.

Original Western blots

Figure 7C

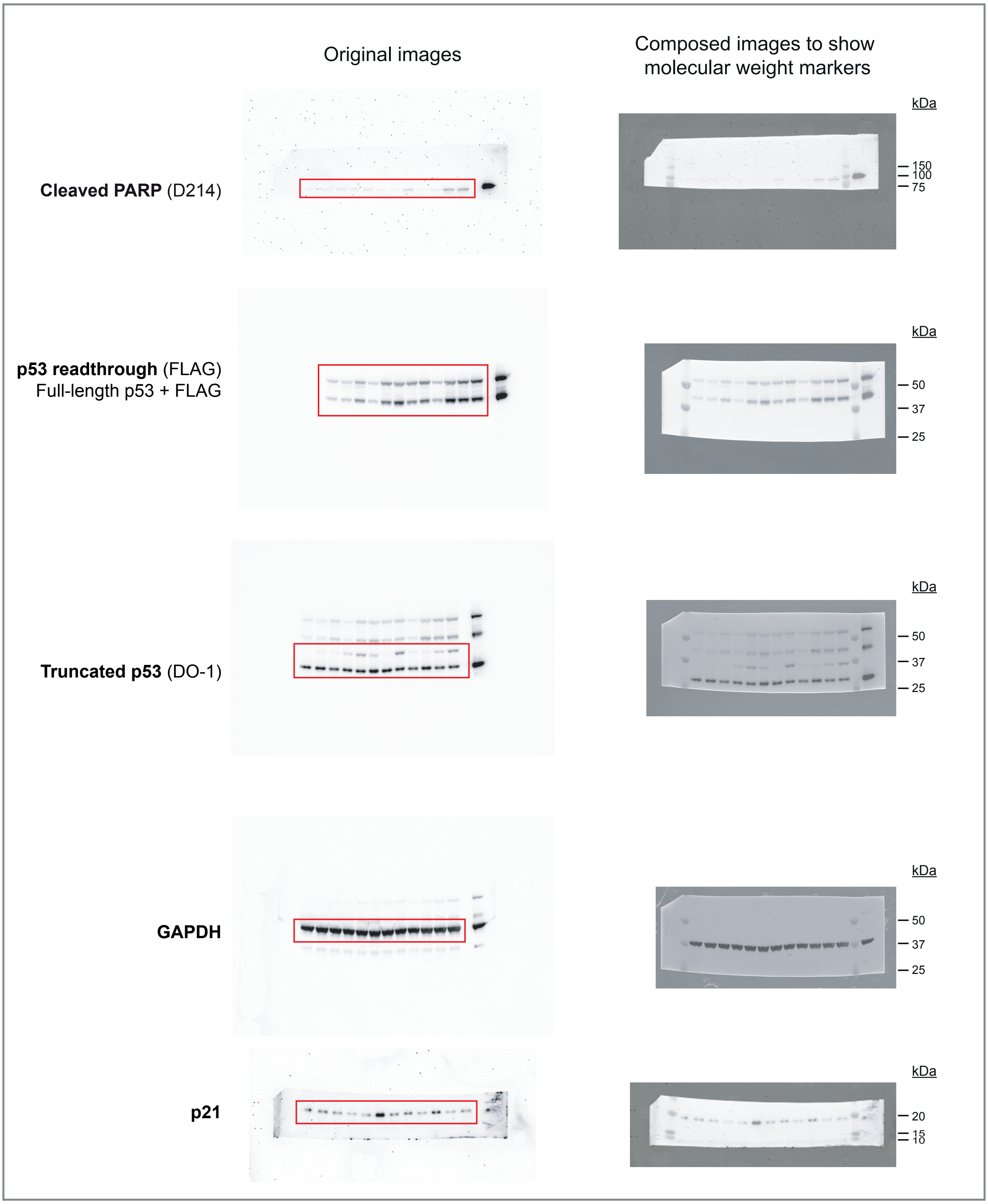

Middle part of the membrane was first blotted with FLAG antibody, washed and blotted with p53 (DO-1) antibody. Thus, some remaining signal from FLAG antibody is observed when developing the p53 (DO-1) antibody. Membrane was then washed and blotted with GAPDH, thus some remaining signal from the two previous antibodies used is observed when developing the GAPDH antibody.

Original Western blots

Figure S2C

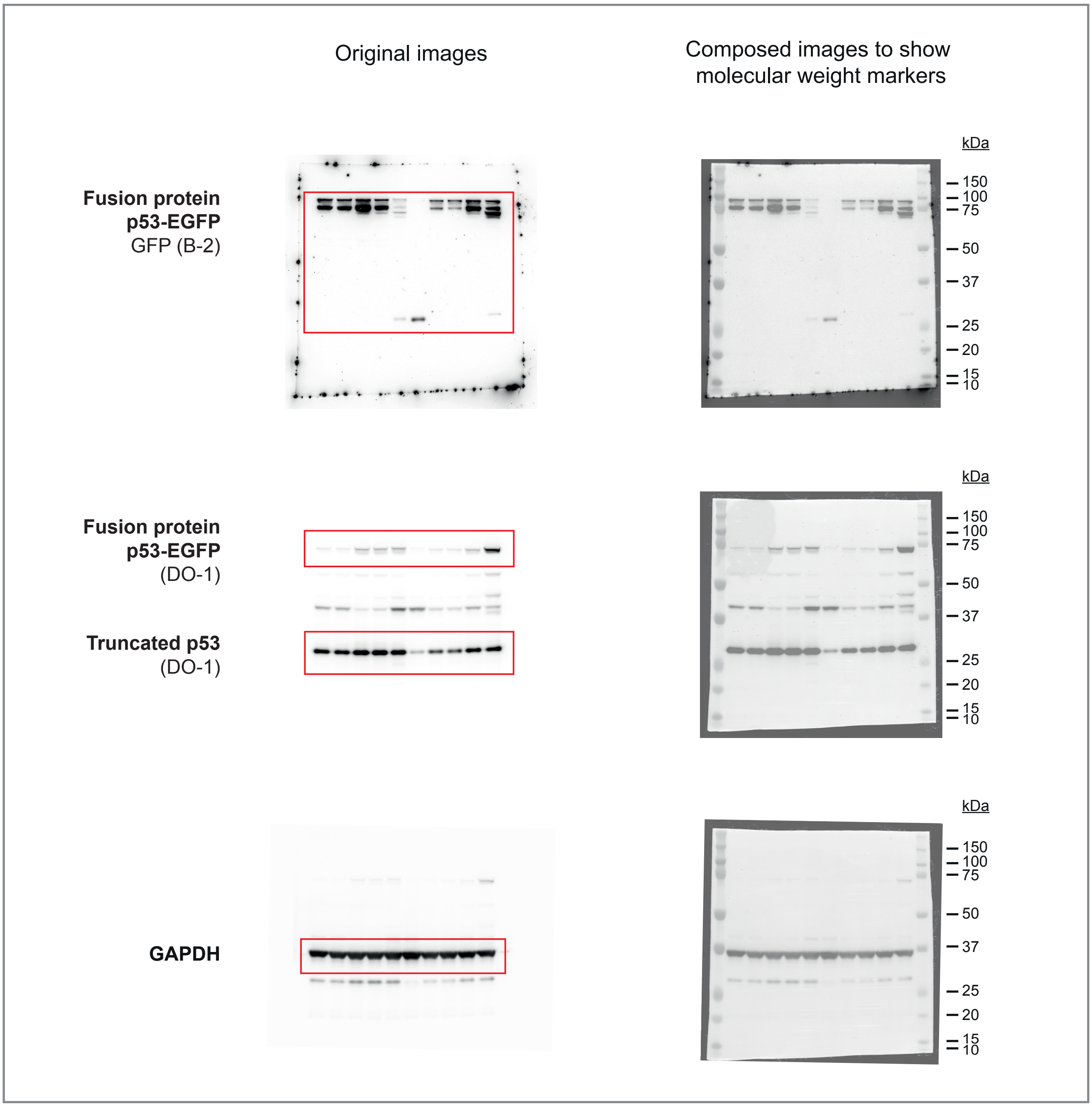

Membrane was first blotted with GFP (B-2) antibody. Then, it was stripped, blocked again and blotted with p53 (DO-1) antibody. Membrane was lastly blotted with GAPDH antibody, thus some remaining signal from the p53 (DO-1) antibody is observed when developing the GAPDH antibody.

Original Western blots

Figure S2D

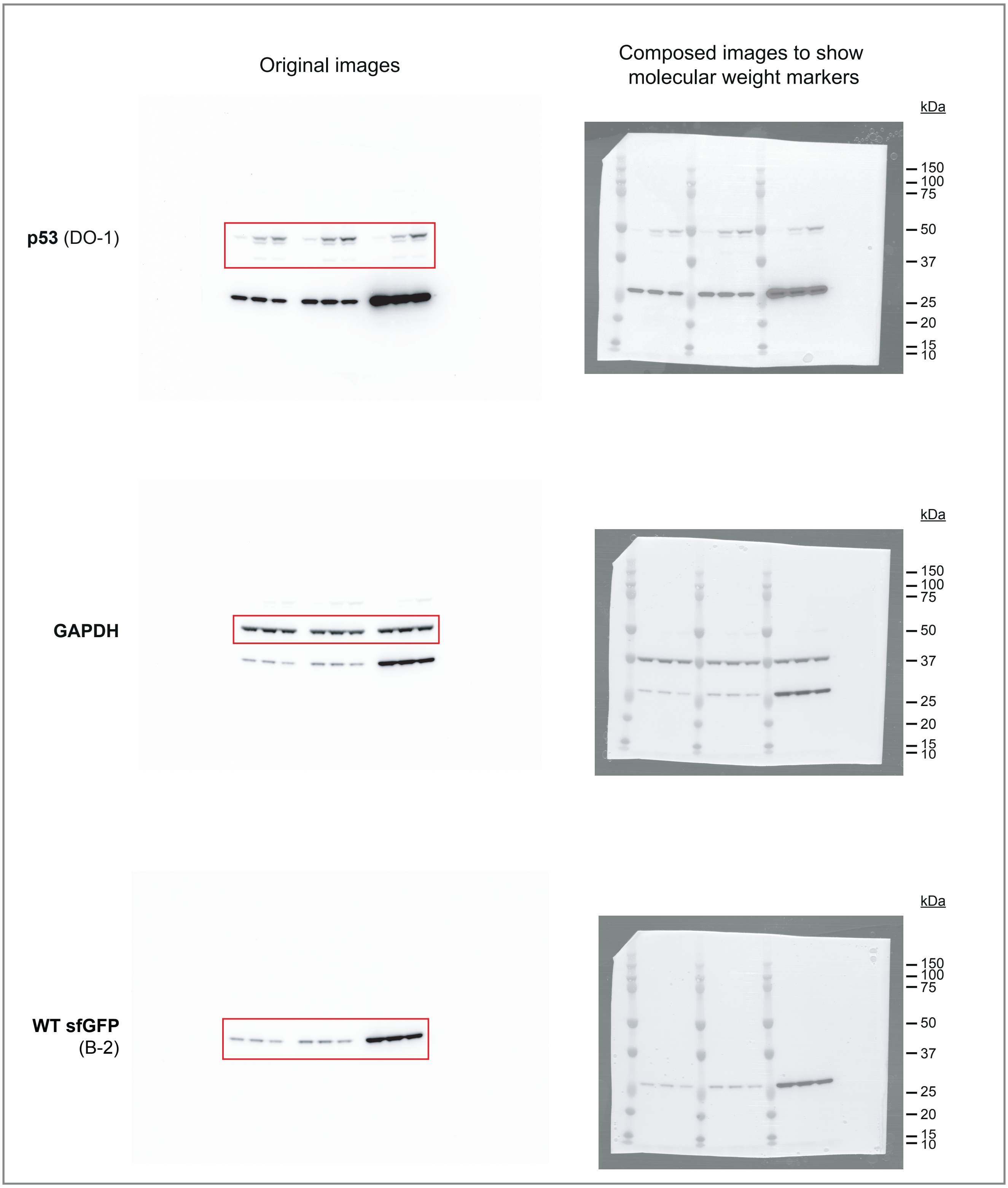

Membrane was first blotted with GFP (B-2) antibody, washed and blotted with p53 (DO-1) antibody, thus the strong band observed below the p53 signal corresponds to WT sfGFP. Membrane was lastly blotted with GAPDH antibody, thus some remaining signal from the two previous antibodies used are observed when developing the GAPDH antibody.

Original Western blots

Figure S2E

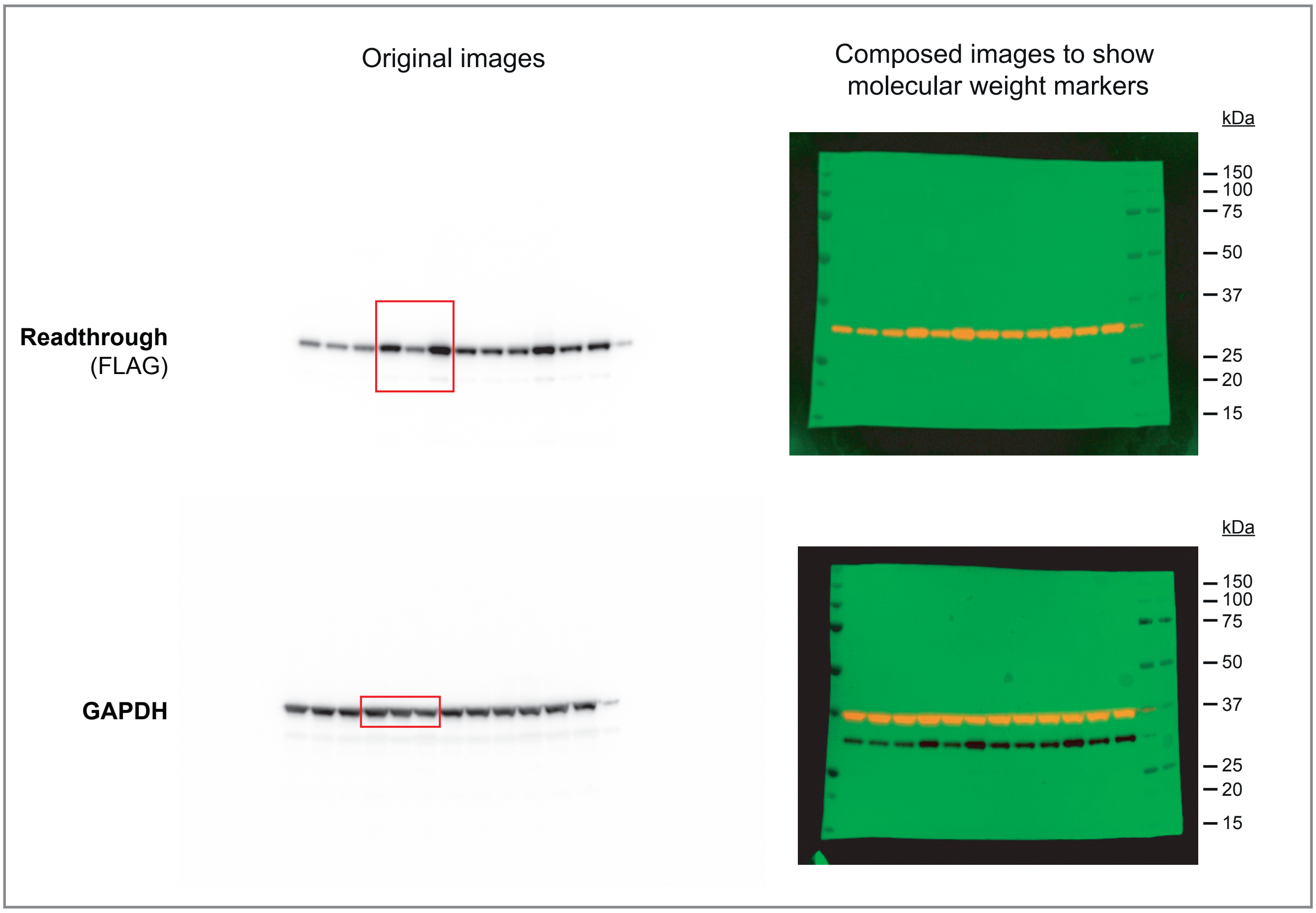

Membrane was first blotted with FLAG antibody, washed and blotted with GAPDH antibody, thus the black signal in the bottom right composed image (for GAPDH) corresponds to FLAG signal and the orange bands correspond to GAPDH.

Original Western blots

Figure S4C

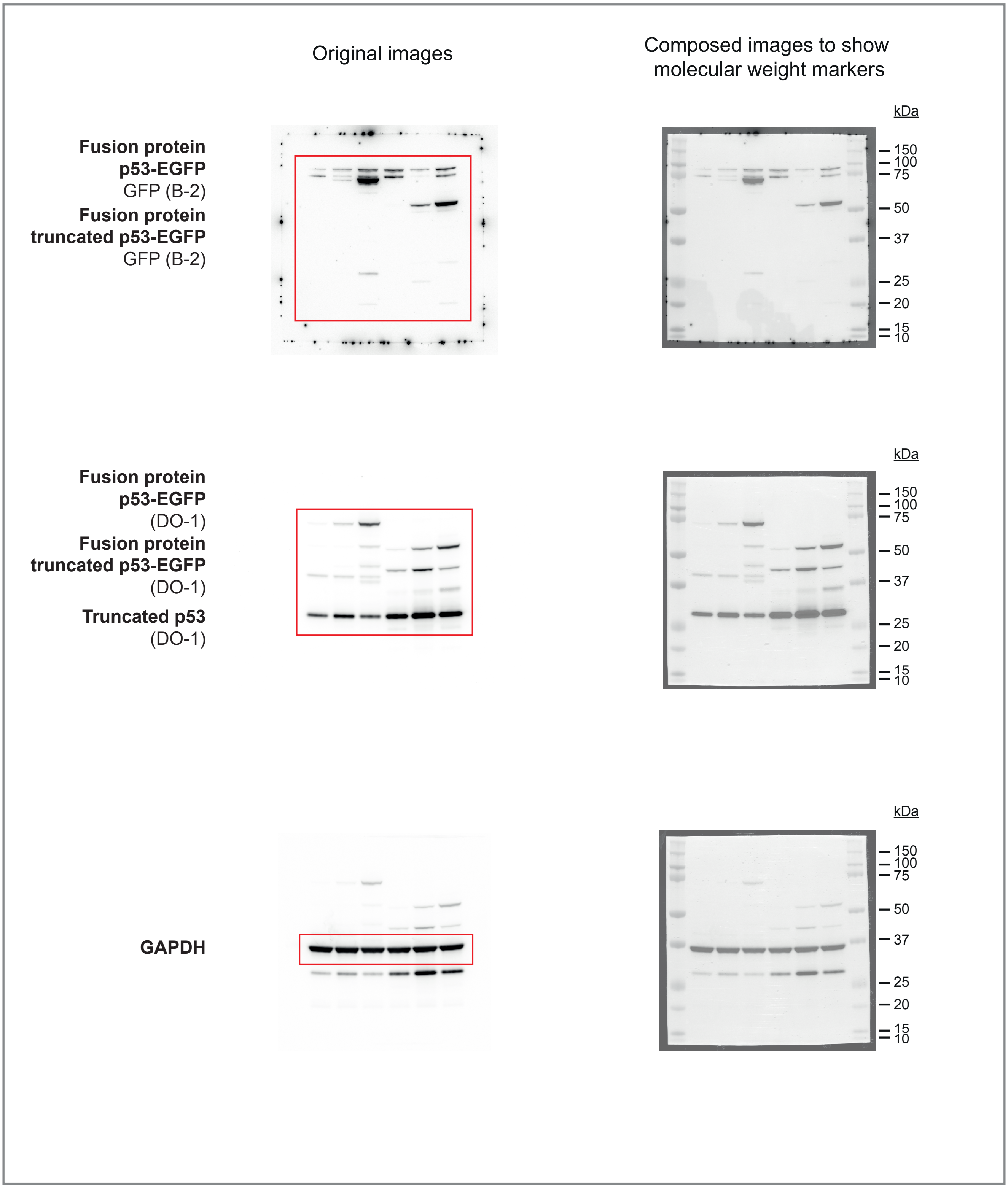

Membrane was first blotted with GFP (B-2) antibody. Then, it was stripped, blocked again and blotted with p53 (DO-1) antibody. Membrane was lastly blotted with GAPDH antibody, thus some remaining signal from the p53 (DO-1) antibody is observed when developing the GAPDH antibody.

Original Western blots

Figure S7A

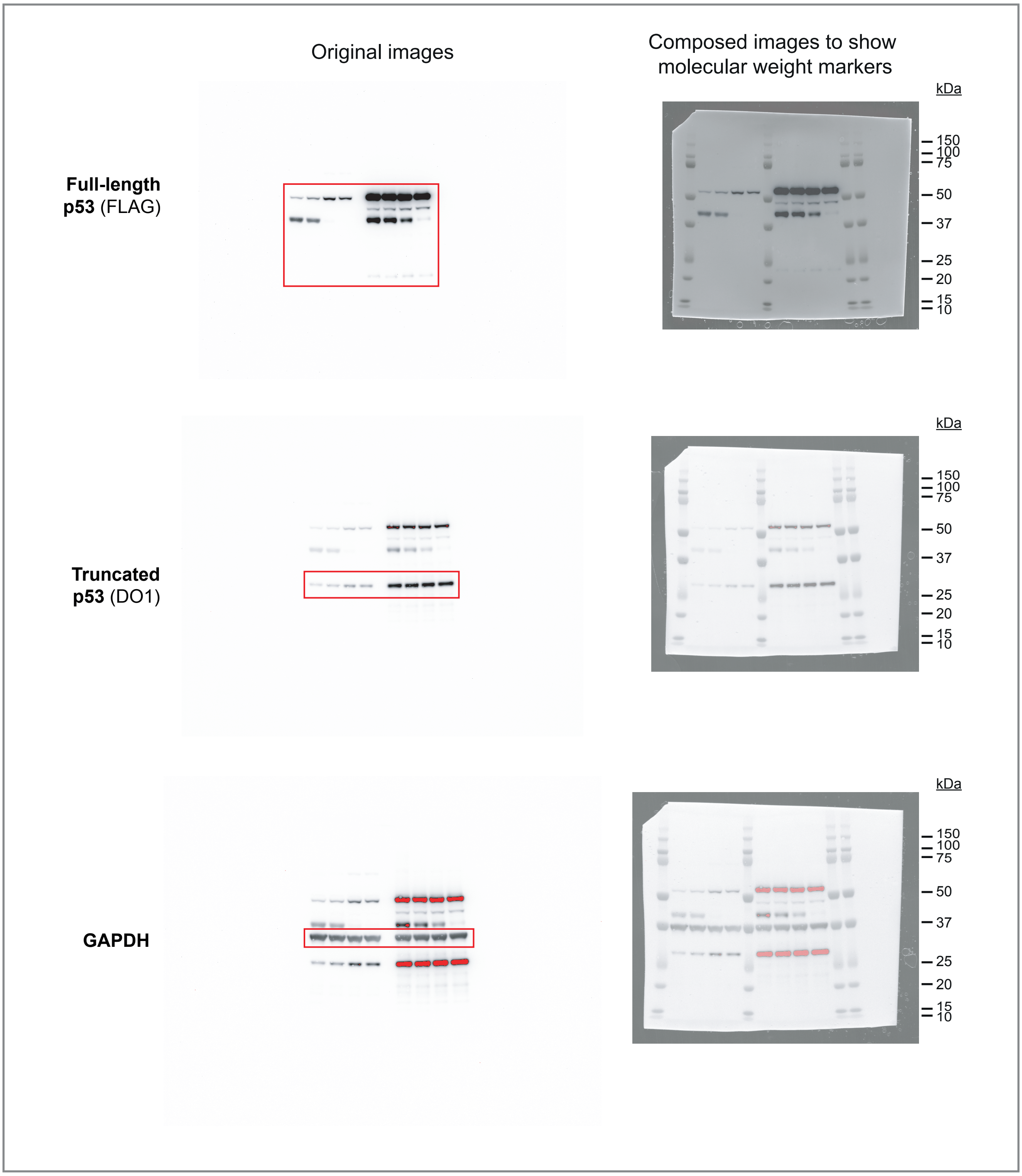

Membrane was first blotted with FLAG antibody, washed and blotted with p53 (DO-1) antibody, thus the bands above the p53 signal belong to FLAG signal. Membrane was lastly blotted with GAPDH antibody, thus some remaining signal from the two previously used antibodies are observed when developing the GAPDH antibody.

Original Western blots

Figure S7B

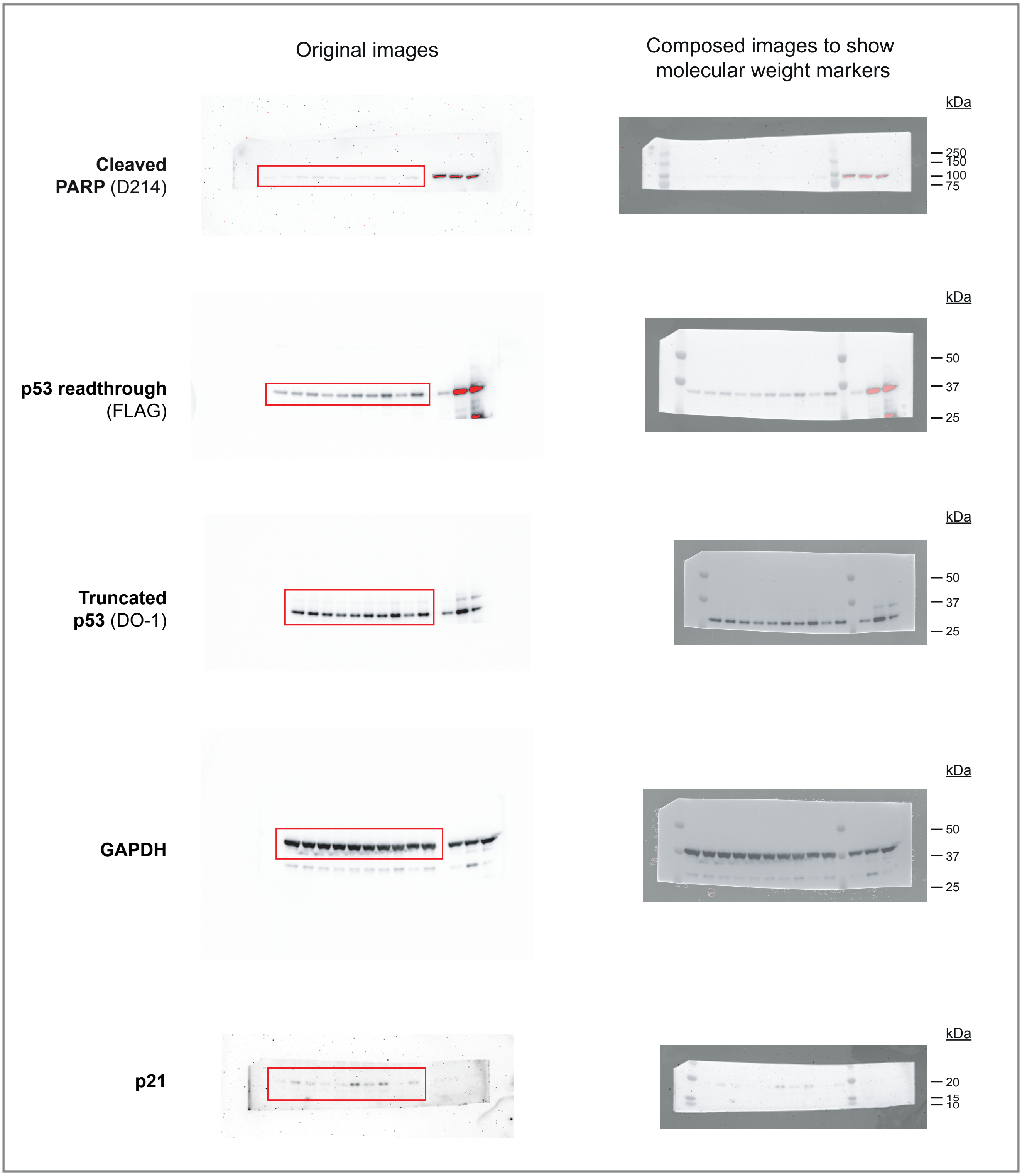

Some extra bands at an angle below the GAPDH signal can be seen due to some protein transfer before repositioning the gel in the membrane before performing the protein transfer.

Middle part of the membrane was first blotted with FLAG antibody, washed and blotted with p53 (DO-1) antibody. Thus, some remaining signal from FLAG antibody is observed when developing the p53 (DO-1) antibody. Membrane was then washed and blotted with GAPDH, thus some remaining signal from the two previous antibodies used are observed when developing the GAPDH antibody.
